# A chronic oral reference dose for hexavalent chromium-induced intestinal cancer†

**DOI:** 10.1002/jat.2907

**Published:** 2013-08-14

**Authors:** Chad M Thompson, Christopher R Kirman, Deborah M Proctor, Laurie C Haws, Mina Suh, Sean M Hays, J Gregory Hixon, Mark A Harris

**Affiliations:** a ToxStrategies, Inc.Katy, TX, 77494, USA; b Summit Toxicology, LLPOrange Village, OH, 44022, USA; c ToxStrategies, Inc.Rancho Santa Margarita, CA, 92688, USA; d ToxStrategies, Inc.Austin, TX, 78731, USA; e Summit Toxicology, LLPAllenspark, CO, 80510, USA

**Keywords:** risk assessment, hexavalent chromium Cr(VI), mode of action, benchmark dose (BMD) modeling, constrained nonlinear regression, cancer reference dose (RfD), intestinal cancer

## Abstract

High concentrations of hexavalent chromium [Cr(VI)] in drinking water induce villous cytotoxicity and compensatory crypt hyperplasia in the small intestines of mice (but not rats). Lifetime exposure to such cytotoxic concentrations increases intestinal neoplasms in mice, suggesting that the mode of action for Cr(VI)-induced intestinal tumors involves chronic wounding and compensatory cell proliferation of the intestine. Therefore, we developed a chronic oral reference dose (RfD) designed to be protective of intestinal damage and thus intestinal cancer. A physiologically based pharmacokinetic model for chromium in mice was used to estimate the amount of Cr(VI) entering each intestinal tissue section (duodenum, jejunum and ileum) from the lumen per day (normalized to intestinal tissue weight). These internal dose metrics, together with corresponding incidences for diffuse hyperplasia, were used to derive points of departure using benchmark dose modeling and constrained nonlinear regression. Both modeling techniques resulted in similar points of departure, which were subsequently converted to human equivalent doses using a human physiologically based pharmacokinetic model. Applying appropriate uncertainty factors, an RfD of 0.006 mg kg^–1^ day^–1^ was derived for diffuse hyperplasia—an effect that precedes tumor formation. This RfD is protective of both noncancer and cancer effects in the small intestine and corresponds to a safe drinking water equivalent level of 210 µg l^–1^. This concentration is higher than the current federal maximum contaminant level for total Cr (100 µg l^–1^) and well above levels of Cr(VI) in US drinking water supplies (typically ≤ 5 µg l^–1^). © 2013 The Authors. *Journal of Applied Toxicology* published by John Wiley & Sons, Ltd.

## Introduction

Exposure to hexavalent chromium [Cr(VI)] has long been recognized to increase the risk of lung cancer among workers in certain industries (IARC, 1990), as well as in rodents via inhalation or intratracheal instillation (Glaser *et al.*, 1986; Steinhoff *et al.*, 1986). Owing to protective reductive mechanisms, ingestion of Cr(VI) was thought to pose relatively little cancer risk (De Flora *et al.*, 1987; De Flora *et al.*, 1997; Febel *et al.*, 2001; Proctor *et al.*, 2002). In fact, Cr(VI) has not been shown to cause a significantly increased cancer risk in the alimentary canal of exposed workers (Gatto *et al.*, 2010). However, a recent 2-year cancer bioassay indicated that chronic exposure to Cr(VI), administered as sodium dichromate dihydrate, caused a dose-dependent increase in intestinal damage and intestinal tumor formation in B6C3F1 mice, but not F344 rats (NTP, 2008b). Subchronic bioassays indicated increased intestinal damage in mice after 90 days of exposure, but without evidence of preneoplastic lesions (NTP, 2007; Thompson *et al.*, 2011b). It is well known that chemicals that cause cytotoxicity and subsequently induce cell proliferation in shorter-term assays are often carcinogenic in longer-term bioassays (Ames *et al.*, 1993; Boobis *et al.*, 2009; Cohen, 2010; Gaylor, 2005). Thus, the disparate outcomes observed in mice and rats suggested that the intestinal tumors observed in mice were the result of chronic mucosal injury with compensatory regenerative hyperplasia.

To investigate the mode of action (MOA) underlying intestinal tumors in mice, a series of studies were conducted to collect biochemical, histological and pharmacokinetic data in the rodent small intestine (see section on “Mode of action for intestinal neoplasms”). Collectively, these studies indicate that Cr(VI) induced early and prolonged (lifetime) intestinal damage and crypt hyperplasia in mice. Despite the increase in crypt hyperplasia, exposure to Cr(VI) for up to 90 days did not induce cytogenetic damage in duodenal crypts cells or increase K-*ras* mutant frequency in duodenal tissues at carcinogenic concentrations (O'Brien *et al.*, 2013). The weight of evidence from the aforementioned studies supports a nonmutagenic MOA based on chronic intestinal wounding of nonproliferative villous tissue, which results in compensatory regenerative crypt hyperplasia and, ultimately, intestinal carcinogenesis (Thompson *et al.*, 2013). Therefore, an oral reference dose (RfD) that is protective of diffuse hyperplasia would also be protective of Cr(VI)-induced intestinal cancer. An RfD based on intestinal irritation has previously deemed protective for other small intestine (SI) carcinogens (Gordon, 2007; US EPA, 2004).

**Figure 1:**
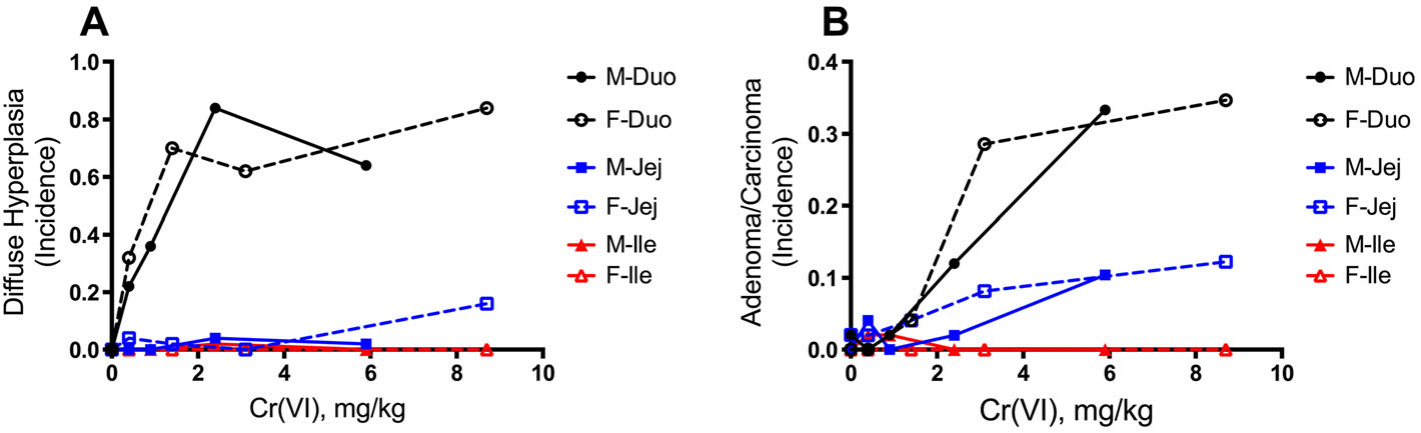
Dose–response of key intestinal lesions in mice from the NTP (2008b) 2-year bioassay. (A) Incidence of diffuse hyperplasia in the duodenum, jejunum and ileum of male and female mice. (B) Combined incidence of adenomas and carcinomas in the duodenum, jejunum and ileum of male and female mice. Duo, duodenum; F, female; Ile, ileum; Jej, jejunum; M, male.

The purpose of this article is to describe the derivation of an RfD that is protective of both cancer and noncancer effects of Cr(VI) in the SI. Dose–response data collected by the National Toxicology Program (NTP) indicate a response gradient for mouse SI hyperplasia and tumor formation, with responses being greatest in the duodenum, moderate in the jejunum, and absent in the ileum (Fig. 1; (NTP, 2008b)). Target tissue chromium concentration data collected from mice indicate that total chromium concentrations in the SI exhibit a strong concentration-dependent gradient that parallels the observed tissue responses in the NTP bioassay, with chromium concentrations being highest in the duodenum, moderate in the jejunum and relatively low in the ileum (Kirman *et al.*, 2012; Thompson *et al.*, 2011b). A rodent physiologically based pharmacokinetic (PBPK) model was used to estimate target tissue doses for Cr(VI) corresponding to applied doses in the NTP 2-year animal bioassay. Because tissue response data were collected from each of the SI sections (duodenum, jejunum, ileum) of male and female mice from four different treatment groups, a robust dose–response data set was generated with as many as 24 data points (four dose groups, two sexes, three intestinal segments per animal), each representing approximately 50 observations. Benchmark dose (BMD) modeling and constrained nonlinear regression (CNR) techniques were used to derive points of departure (PODs) that were subsequently converted to human equivalent doses using a human PBPK model (Kirman *et al.*, 2013). Applying standard uncertainty factors allowed for the derivation of a chronic RfD and drinking water equivalent level. This Cr(VI) risk assessment is technically and scientifically more refined than previous assessments, because it: (1) uses MOA information to identify critical precursor endpoints for dose–response analysis; (2) uses MOA information to inform appropriate low-dose extrapolation methods; (3) employs rodent and human PBPK models to quantify target tissue dose and extrapolate between species and across dose levels; and (4) applies multiple quantitative dose–response modeling techniques. Further, the methods and approaches used in this assessment are consistent with US EPA guidance on best risk assessment practices (US EPA, 2005, 2006, 2011, 2012).

## Methods

### Data selection

For dose–response modeling of diffuse hyperplasia and tumor formation, male and female data were combined because visual examination (see Fig. 1; NTP, 2008b) and statistical analysis revealed no evidence of sex differences in response to Cr(VI). Specifically, logistic regression was conducted using each response variable as the dependent variable, and dose, sex, and the dose × sex interaction as independent variables. The main effect of sex and the dose × sex interaction effect were assessed for each of the six combinations of response (adenoma/carcinoma or hyperplasia) and segment (duodenum, jejunum or ileum). The results for each effect were then combined into a composite test. Across the six combinations there was no main effect of sex (χ^2^(6) = 6.84, *P* = 0.34), and no dose × sex interaction effect (χ^2^(6) = 7.21, *P* = 0.30); i.e., the effects of dose did not vary significantly across the sexes.

**Figure 2:**
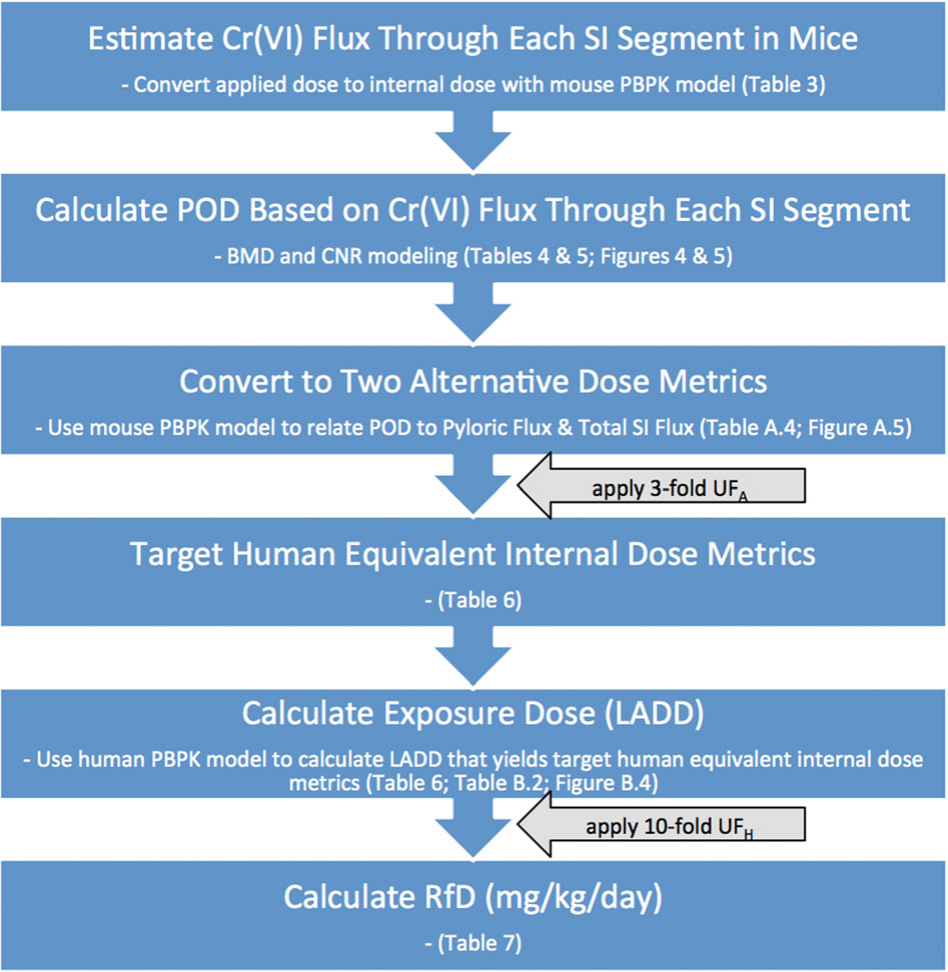
Process chart for derivation of RfD. LADD, lifetime average daily doses; POD, points of departure; RfD, reference dose.

Although the US EPA has historically assessed dose–response data for male and female animals separately, combining data across sex is consistent with recent BMD guidelines that state, “Datasets that are statistically and biologically compatible may be combined prior to dose–response modeling, resulting in increased confidence, both statistical and biological, in the calculated BMD” (US EPA, 2012). Using data for both sexes increases the number of observations for dose–response modeling, which allows for better characterization of the dose–response relationship. In addition to using data from both male and female mice, it was also possible to use data from each intestinal segment because the NTP study provided incidence data in the duodenum, jejunum and ileum of each animal. With the availability of a rodent PBPK model (Kirman *et al.*, 2012), it was possible to predict the dose metric for chromium in each intestinal segment (see section on ‘Hazard identification’). The overall process for RfD derivation is shown in Fig. 2.

### Dose–response modeling

Applied study doses for relevant endpoints were converted to internal dose metrics in target tissues of mice using a previously published PBPK model (Kirman *et al.*, 2012). For each study dose, the PBPK model was used to estimate the internal dose of Cr(VI) entering each intestinal segment (duodenum, jejunum and ileum) (see section on ‘Dose metric selection’ and Appendix A). Dose–response modeling for adverse effects was conducted using US EPA's BMD Software (BMDS) v.2.3, using the suite of dichotomous models as well as the dichotomous-Hill model. Benchmark response (BMR) values of 5% and 10% extra risk were used to obtain BMD (BMD_x_) values, along with their corresponding 95% lower confidence limit (BMDL_x_), per US EPA recommendations (US EPA, 2012). The slopes were restricted to ≥ 1, which is done to prevent the estimated dose–response curve from taking on a biologically implausible very steep slope as the dose approaches zero. Model fits were judged using criteria such as *P*-values, scaled residuals, Akaike information criterion, parsimony and visual inspection. In addition to BMD modeling, CNR was conducted using GraphPad Prism 6 for Mac (http://www.graphpad.com) in an effort to characterize the relationship between dose, incidence and progression of disease (hyperplasia, adenoma, carcinoma) with a single Hill model: 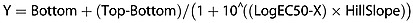
where, 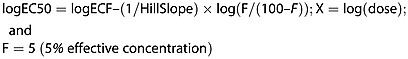
Models were constrained by sharing model parameters such as Hill slope and maximum response. The effective concentration (EC) values and their 95% lower confidence limits (ECL) (computed using GraphPad) were compared to BMD and BMDL values (computed using BMDS). BMDL and ECL values based on internal doses were converted to human equivalent doses (HEDs) using a previously published human PBPK model for the disposition of ingested chromium (Kirman *et al.*, 2013). All PBPK modeling was performed in Advanced Continuous Simulation Language Extreme and its add-in for Microsoft Excel (asclX version 3; Aegis TG; http://www.acslx.com).

An RfD value was derived as follows. The mouse POD was first divided by the uncertainty factor (UF) for interspecies variation (UF_A_) for two reasons: (1) this permits the calculation of a human equivalent POD value (calculated as mouse POD/UF_A_), which can then be used to support a margin-of-exposure analysis, and (2) application of the UF_A_ term likely ensures that the interspecies extrapolation step is performed in a region where linear toxicokinetics are predicted in both species. The remaining UF values were then applied to HEDs corresponding to the mouse POD/UF_A_ as depicted in the equation below: 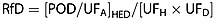
where,

RfD = (mg kg^–1^ day^–1^)
POD = Point of departure (expressed in terms of internal dose)
UF_A_ = uncertainty factor for interspecies variation (unitless)
UF_H_ = uncertainty factor for intraspecies variation (unitless); and
UF_D_ = uncertainty factor for database deficiencies (unitless).

### Results

#### Hazard identification

This study focuses on the intestinal toxicity and carcinogenicity of Cr(VI) following ingestion, and thus does not discuss other effects of Cr(VI) outside the SI. To date, the most robust study of the oral toxicity and carcinogenicity of Cr(VI) was conducted by the NTP (NTP, 2008b; Stout *et al.*, 2009). The only lesion observed in the rat SI was histiocytic infiltration. In contrast, mice exhibited histiocytic infiltration and diffuse hyperplasia, and developed adenomas and carcinomas late in life. The NTP study authors concluded that the meaning of histiocytic infiltration was uncertain (NTP, 2008b), and our own MOA analysis did not consider this a critical effect (Thompson *et al.*, 2013). It is also notable that 90-day Cr(VI) studies in rats and mice revealed diffuse hyperplasia in the duodena of mice but not rats (NTP, 2007). It is well accepted that chemicals that induce cytotoxicity and cell proliferation in shorter-term bioassays are often carcinogenic in longer-term bioassays (Ames *et al.*, 1993; Boobis *et al.*, 2009; Cohen, 2010; Gaylor, 2005). We recently showed that Cr(VI) concentrations carcinogenic in mice induce villous cytotoxicity and crypt cell proliferation after only 7 days of exposure (Thompson *et al.*, 2011b). As outlined in the following section, recent studies strongly support that diffuse hyperplasia is a major risk factor (i.e., key event) in the development of intestinal cancer.

#### Mode of action for intestinal neoplasms

To investigate the MOA for intestinal carcinogenesis, a series of studies were conducted to collect histological, biochemical, toxicogenomic and pharmacokinetic data in the rodent SI (Kirman *et al.*, 2012; Kirman *et al.*, 2013; Kopec *et al.*, 2012a, 2012b; O'Brien *et al.*, 2013; Proctor *et al.*, 2012; Thompson *et al.*, 2011a, 2011b, 2012a, 2012b, 2012c). These data were evaluated along with other relevant literature (including the NTP study findings) to develop a MOA for intestinal carcinogenesis (Thompson *et al.*, 2013). The overall weight of evidence supports a cytotoxic MOA with the following key events: absorption of Cr(VI) from the intestinal lumen. villous cytotoxicity. compensatory crypt hyperplasia, and crypt cell mutagenesis (expansion of spontaneous mutations in the crypt cells as a consequence of the constant proliferative pressure).

Table 1 summarizes the concentrations at which significant changes in endpoints relevant to the MOA occurred in the 90-day drinking water study by Thompson *et al.* (2011b). At ≥ 5 mg l^–1^ Cr(VI), there were significant increases in duodenal chromium levels. At these same concentrations, significant changes in the GSH/GSSG ratio (a measure of redox status) were observed. Concentrations ≥ 20 mg l^–1^ Cr(VI) were accompanied by large increases in the number of mRNA transcripts that were significantly altered, as well as signs of cytoplasmic vacuolization in the intestinal villi. At ≥ 60 mg l^–1^ Cr(VI) (i.e., carcinogenic concentrations in the NTP 2-year bioassay), crypt cell proliferation was increased. Importantly, cytogenetic damage was not observed in duodenal crypts at any dose, nor were there any Cr(VI)-related increases in *K-ras* codon 12 GAT mutant frequency (O'Brien *et al.*, 2013). Because *K-ras* codon 12 GAT mutant frequency has been shown to be a reporter gene for mutations occurring in other oncogenes (Parsons *et al.*, 2012), the absence of Cr(VI)-induced increases in *K-ras* codon 12 GAT mutant frequency further supports a nonmutagenic MOA. Because the intestinal stem cells reside in the crypts below the mucosal surface, the apparent absence of toxicity and genetic damage in crypt cells following subchronic exposure to carcinogenic concentrations of Cr(VI) indicates that the intestinal tumors arose from chronic tissue damage and regenerative hyperplasia, rather than from direct interaction with DNA of crypt stem cells.

**Table:** Summary of mode of action study findings in mice exposed to Cr(VI)

Sodium dichromate dihydrate (mg l^–1^)	0	0.3^a^	4^a^	14	60	170	520
Cr(VI) (mg l^–1^)	0	0.1^a^	1.4^a^	5	20	60	180
Cr in duodenum	–	–	–	+	+	+	+
Redox changes	–	–	–	+	+	+	+
Gene changes	–	–	–	–	+	+	+
Villus toxicity	–	–	–	–	+	+	+
Crypt proliferation	–	–	–	–	–	+	+
Crypt cytogenetic damage	–	–	–	–	–	–	–
K-*ras* mutations	–	–	–	–	–	–	–
Preneoplastic lesions	–	–	–	–	–	–	–

+ indicates doses where effects differed significantly from control; –, indicates no effect was observed.

a Cr(VI) concentrations not included in the National Toxicology Program studies.

**Figure 3:**
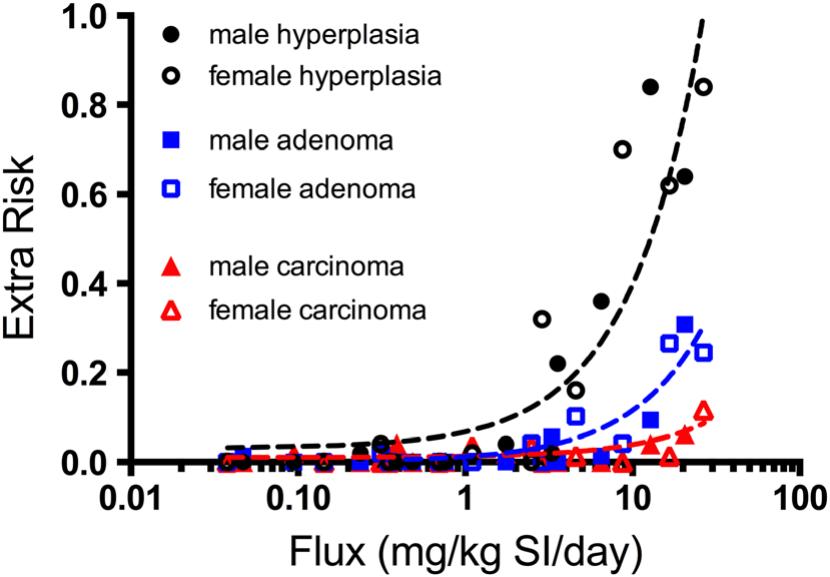
Dose–response of key intestinal endpoints from the small intestines of mice in the NTP (2008b) 2-year bioassay. Filled and open shapes represent data from male and female mice, respectively. The *x*-axis is expressed in terms of flux (i.e., the mg of Cr(VI) estimated to pass through each intestinal segment per day) (see text for details). The lines represent linear regression (*x* is log scale) through combined incidence. These plots are not used for quantitative dose–response modeling, but rather to show the progression of hyperplasia, adenomas and carcinomas. SI, small intestine.

Figure 3 shows the dose–response for intestinal endpoints in male and female mice from the NTP study on an internal dose basis (described in the section on ‘Dose metric selection’). Importantly, the term diffuse hyperplasia in the NTP study included both damage to villi and crypt proliferation. Clearly, intestinal diffuse hyperplasia occurred at lower doses (i.e., preceded) than did tumorigenic responses. Because intestinal diffuse hyperplasia is a precursor to tumor formation, preventing diffuse hyperplasia should preclude increased tumor formation in the intestine. Thus, an oral RfD that is protective of intestinal diffuse hyperplasia would also be protective of cancer.

#### Dose–response analysis

##### Critical effect selection

Diffuse hyperplasia and tumor formation data from the NTP 2-year bioassay were selected for dose–response analysis (NTP, 2008b). Table 2 summarizes the dose–response data set recently used to model these two endpoints based on applied dose (i.e., mg kg^–1^ bodyweight) (US EPA, 2010). However, with the availability of newly developed PBPK models, it was possible to assign the incidence data for diffuse hyperplasia and tumors to each intestinal segment (i.e., duodenum, jejunum, ileum). In doing so, a far more robust dose–response data set (24 treatment groups spanning a range of nearly three orders of magnitude) was generated as compared to that based on administered dose to a single sex (i.e., four treatment groups spanning approximately one order of magnitude).

**Table 2:** Dose–response data sets for mouse small intestine effects using applied dose (US EPA, 2010)

Diffuse hyperplasia					Adenomas/carcinomas				
Segment	Sex	(mg kg^–1^ day^–1^)^a^	n	Hyperplasia	Segment	Sex	(mg kg^–1^ day^–1^)^a^	n	Tumor^b^
d	f	0	50	0	d, j, i	m	0	49	1
d	f	0.38	50	16	d, j, i	m	0.38	49	3
d	f	1.4	50	35	d, j, i	m	0.91	49	2
d	f	3.1^c^	50	31	d, j, i	m	2.4	50	7
d	f	8.7^c^	50	42	d, j, i	m	5.9	48	20

d, duodenum; f, female; i, ileum; j, jejunum; m, male.

a Based on applied dose (mg Cr(VI) per kg bodyweight per day).

b Based on combined incidence of adenomas and carcinomas.

c Data points were dropped to achieve benchmark dose model fits (see text for discussion).

Table 3 shows the incidence data for diffuse hyperplasia and tumor formation (all incidence data are from the NTP 2-year bioassay) assigned to the predicted flux of Cr(VI) into each intestinal segment. The number of observations for hyperplasia and tumors differs, because, consistent with the approach used in US EPA (2010), we excluded animals that died before the appearance of the first intestinal tumor (typically one or two animals per treatment group). [Poly-k adjustments were not used because: (1) Cr(VI) had no effect on survival (NTP, 2008b); (2) US EPA did not use a poly-k adjustment in their risk assessment of Cr(VI) (US EPA, 2010); and (3) the primary effect of concern was non-neoplastic (i.e., diffuse hyperplasia).] Because male and female mice in each treatment group had unique internal dose metrics for each intestinal segment, the sample size (*n*) for each observation was 50, except in cases where animals died prematurely. The *n* for the control groups (i.e., zero internal dose) is the total number of intestinal segments (three per animal) for male and female control mice combined. It is immediately apparent in Table 3 that the segment with the lowest flux (i.e., ileum) characterizes the low end of the dose–response curve, and the tissue with the highest flux (i.e., duodenum) characterizes the upper end of the dose–response curve. These data are consistent with the NTP study findings of the rank of adverse effects in the intestine (duodenum > jejunum > > ileum) (NTP, 2008b; Stout *et al.*, 2009), as well as the chromium tissue burden measured in each intestinal segment (Kirman *et al.*, 2012; Thompson *et al.*, 2011b).

**Table 3:** Dose–response data set for mouse small intestine effects using internal dose

Hyperplasia					Tumor incidence					
Segment	Sex	Flux (mg kg^–1^ SI day^–1^)^a^	n	Hyperplasia	Segment	Sex	Flux (mg kg^–1^ SI d^–1^)^a^	N	Adenoma	Carcinoma
d, j, i	m, f	0	300	0	d, j, i	m, f	0	294	1	1
i	f	0.0377	50	0	i	f	0.0377	50	0	0
i	m	0.0469	50	0	i	m	0.0469	49	1	0
i	m	0.0943	50	0	i	m	0.0943	49	0	1
i	f	0.143	50	0	i	f	0.143	49	0	0
i	m	0.236	50	1	i	m	0.236	50	0	0
j	f	0.312	50	2	j	f	0.312	50	1	0
i	f	0.351	50	0	i	f	0.351	49	0	0
j	m	0.389	50	0	j	m	0.389	49	0	2
i	m	0.485	50	0	i	m	0.485	48	0	0
i	f	0.701	50	0	i	f	0.701	49	0	0
j	m	0.760	50	0	j	m	0.760	49	0	0
j	f	1.10	50	1	j	f	1.10	49	0	2
j	m	1.75	50	2	j	m	1.75	50	0	1
j	f	2.48	50	0	j	f	2.48	49	2	2
d	f	2.88	50	16	d	f	2.88	50	0	0
j	m	3.29	50	1	j	m	3.29	48	3	2
d	m	3.56	50	11	d	m	3.56	49	0	0
j	f	4.58	50	8	j	f	4.58	49	5	1
d	m	6.50	50	18	d	m	6.50	49	1	0
d	f	8.69	50	35	d	f	8.69	49	2	0
d	m	12.8	50	42	d	m	12.8	50	5	2
d	f	16.6	50	31	d	f	16.6	49	13	1
d	m	20.5	50	32	d	m	20.5	48	15	3
d	f	26.6	50	42	d	f	26.6	49	12	6

d, duodenum; f, female; i, ileum; j, jejunum; m, male; SI, small intestine.

a Based on mg Cr(VI) per kg of small intestine (SI) segment per day.

These values are also reported in Appendix Table A.3.

##### Dose metric selection

The selection of an appropriate dose measure requires careful consideration of the MOA (US EPA, 2006). A number of candidate internal dose measures are available for assessing the dose–response relationship for small intestinal tumors and diffuse hyperplasia in the mouse, including those for different valence states [Cr(III), Cr(VI), total Cr]. Using a published PBPK model (Kirman *et al.*, 2012), the Cr(VI) concentration in the intestinal lumen and Cr(VI) flux into tissues may be predicted for mice and used as internal dose measures. With respect to valence state, dose measures for Cr(III) and total Cr are not considered useful, for two reasons. First, based on the proposed MOA for mouse SI tumors (Thompson *et al.*, 2013), Cr(III) is not causally related to the formation of tumors. Second, measures of Cr(III) and total Cr do not appear to be useful for predicting tumor response, because model predictions for the Cr(III) tissue doses (for which no neoplastic or non-neoplastic intestinal pathology was observed) from NTP's bioassay for chromium picolinate (NTP, 2008a) overlap the dose regions associated with measurable effects in mice from NTP's Cr(VI) bioassay (NTP, 2008b) (data not shown). For these reasons, internal dose measures for Cr(VI) were selected for dose–response assessment.

For tumors in the mouse SI, potential candidate dose measures include those for Cr(VI) concentration (e.g., in the lumen or tissue of the small intestines) or Cr(VI) flux (e.g., Cr(VI) leaving the stomach lumen or entering into the duodenum, jejunum, and ileum). In addition to MOA considerations, selection of an appropriate dose measure should consider confidence in the PBPK model predictions. Greater confidence is placed on intestinal tissue dose predictions, because these are underpinned by measurements of total Cr in intestinal tissue (Kirman *et al.*, 2012), while corresponding measurements in gastrointestinal lumen are not available. We consider Cr(VI) tissue flux, defined as the amount (mg) of Cr(VI) entering intestinal tissue sections from the gastrointestinal lumen (normalized to per kg intestinal tissue per day), to be the best available dose metric for risk assessment, for the following reasons. First, tissue flux estimates are not affected by subsequent processes (intracellular reduction, transfer to blood, intestinal tissue sloughing) that are more uncertain in the model, and therefore can be predicted with greater confidence than tissue concentration in the PBPK model. Second, although total Cr tissue concentration data are available for rodents (Kirman *et al.*, 2012), such data are not available for humans. Because the SI serves as the primary site of absorption, estimates of Cr(VI) flux can be linked to measures of total Cr in human tissues and urinary excretion (see text below). As shown in Fig. 3, visual inspection of the NTP mouse SI data indicate that the tissue flux of Cr(VI) into each intestinal segment (duodenum, jejunum, and ileum) indeed provides an excellent dose–response concordance of the hyperplasia and tumor response in the mouse SI. Moreover, the plots clearly support that male and female mice responded similarly to Cr(VI) as was indicated by responses on a mg kg^–1^ bodyweight basis in Fig. 1.

##### Dose–response modeling

##### Benchmark dose modeling

BMD modeling was conducted on three endpoints for mouse SI from the NTP study: (1) incidence of diffuse hyperplasia; (2) incidence of adenomas; and (3) incidence of carcinomas. For modeling hyperplasia data, we omitted the jejunum for the following reasons. First, unlike tumor incidence that was assessed grossly across each intestinal segment, hyperplasia incidence was assessed microscopically by a single 5 µm biopsy taken at the approximate midpoint of each intestinal segment. The duodenum and ileum are each ∼ 9 cm long whereas the jejunum is ∼ 19 cm long—implying that the biopsy taken in the jejunum may not accurately reflect hyperplasia in the proximal jejunum. Second, pharmacokinetic data indicate that there is a proximal-to-distal decrease in intestinal tissue Cr concentrations between the duodenum and ileum (i.e., within the jejunum) (Kirman *et al.*, 2012; Thompson *et al.*, 2011b). Considering that the modeled flux values in the jejunum do not account for this gradient and that a single 5 µm section along a 19 cm tube was used to score hyperplasia in the jejunum, there is considerable uncertainty with regard to incidence data of jejunal hyperplasia. In contrast, the high Cr tissue concentrations in the duodenum were associated with hyperplasia and tumor formation, and the very low Cr tissue concentrations in the ileum were not associated with hyperplasia or tumors. Therefore, the dose–response modeling of hyperplasia was conducted without the jejunal data. Because biopsies for hyperplasia at the midpoint of the jejunum may underestimate the incidence of hyperplasia in the proximal jejunum, omission of these data from the dose–response modeling may be viewed as health protective because inclusion of jejunal data would only serve to increase the predicted POD values, albeit with poorer model fits.

**Figure 4:**
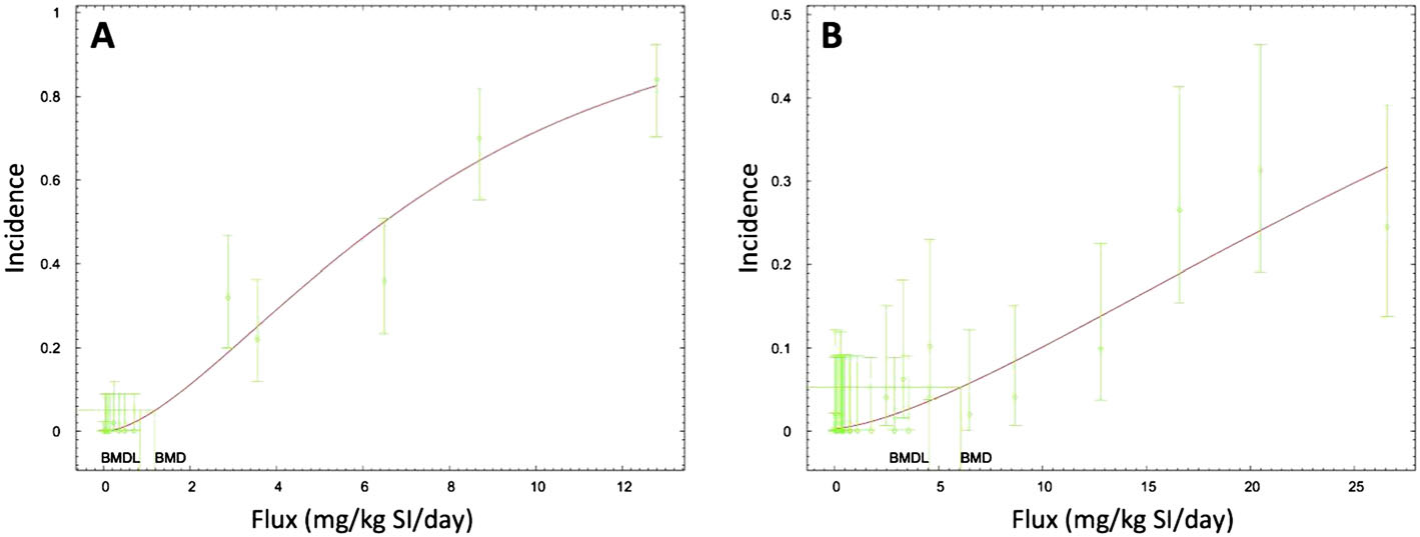
BMD modeling of the hyperplasia incidence (A) and adenoma incidence (B) in the mouse small intestine as a function of internal dose (mg Cr(VI) per kg SI per day). Incidence data are from NTP (2008b). BMD, benchmark dose; BMDL, benchmark dose values and their 95% lower confidence limits; SI, small intestine.

BMD modeling with the duodenal and ileal data resulted in good fitting models with respect to *P*-value (i.e., > 0.1); however, the scaled residuals for most all models were outside EPA's recommended range of ± 2. This indicates that although the models fit the data, they may not fit optimally near the BMD. Notably, the scaled residual value for best fitting model (namely 2.3) only slightly exceeded this cutoff. Nevertheless, we determined that the scaled residuals were acceptable at a BMR of 5% (i.e., lower down the dose–response curve). Selecting a lower BMR is justifiable because the BMR is still within the observable range of data (US EPA, 2012), and is furthermore health protective. This resulted in a BMDL_05-flux_ value of 0.84 mg kg^–1^ SI day^–1^ (Table 4; Fig. 4A).

**Table 4:** Summary of BMD model fits for diffuse hyperplasia and intestinal tumors

Endpoint	Segment	Sex	BMD_05-flux_ (BMD_10-flux_)	BMDL_05-flux_ (BMDL_10-flux_)	*P*-value^a^	Doses^b^ drop/tot
Diffuse hyperplasia	d, i	m, f	1.2	0.84	0.16	3/16
			(1.8)	(1.4)		
Adenomas	d, j, i	m, f	6.1	4.5	0.10	0/24
			(10.1)	(8.3)		
Carcinomas	d, j, i	m, f	19.7	16.4	0.13	0/24
			(26.2)	(21.8)		

d, duodenum; f, female; i, ileum; j, jejunum; m, male.

a *P* ≥ 0.1 indicates good model fit.

b Number of dropped high doses/number of total possible doses (not including control); high doses were dropped (sequentially) until *P*-value for model fit was ≥0.1.

As mentioned above, tumors were assessed across the entire length of each intestinal segment, and thus tumor incidence data for the jejunum were modeled together with the duodenum and ileum. Consistent with the plots in Fig. 3, the BMDL_05-flux_ values for adenomas were lower than for carcinomas (e.g., 4.5 vs. 16.4 mg kg^–1^ SI day^–1^; Table 4). These findings are consistent with the notion that intestinal adenocarcinomas are thought to be the result of a progression from adenomas to carcinomas (Grady and Carethers, 2008; Greaves, 2012). Notably, modeling the combined incidence for adenomas and carcinomas resulted in models with *P*-values for model fit less than 0.1 (data not shown). The BMD plot for the incidence of adenomas is shown in Fig. 4(B).

##### Constrained nonlinear regression

**Figure 5:**
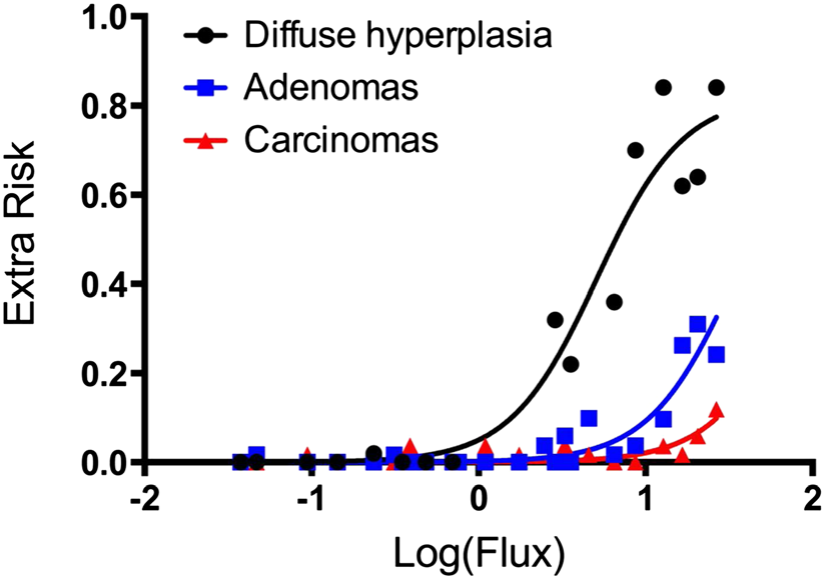
Constrained nonlinear regression of incidence data in NTP 2-year bioassay. Effective concentration values and their 95% lower confidence limits were computed for hyperplasia, adenomas and carcinomas by constraining the models to share the Hill slope and maximal response. Corresponding effective concentration values and their 95% lower confidence limits, Hill slopes and *R*^2^ values are shown in Table 5. For tumors, data from all SI segments were used whereas only the duodenum and ileum were used for hyperplasia (see text).

In addition to BMD modeling, CNR was conducted to obtain EC_05_, EC_10_, ECL_05_ and ECL_10_ values for diffuse hyperplasia and tumor formation using a Hill model. This analysis is not meant to supplant the BMD modeling results, but rather to assess their validity using different modeling approaches. Specifically, CNR allows for finding model solutions to multiple data sets simultaneously by sharing information from each data set. In this way, the dose–response relationships for hyperplasia, adenoma and carcinoma can be characterized using a single model. By sharing parameters, CNR modeling assumes that the incidence of small intestinal tumors in the low-dose region is proportional to the incidence of diffuse hyperplasia. We compared the ECL values for hyperplasia (in duodenum and ileum), adenomas (in all segments) and carcinomas (in all segments) by constraining the models to share the same Hill slope and maximal response parameters (Fig. 5). Overall, the ECL values were in remarkably close agreement with the BMDL values reported in Table 4. For example, the ECL_05_ values for hyperplasia, adenomas and carcinomas were respectively 0.6, 4.2 and 9.4 mg kg^–1^ SI day^–1^ (Table 5), which are similar to the BMDL_05_ values of 0.8, 4.5 and 16.4 mg kg^–1^ SI day^–1^ (Table 4).

**Table 5:** EC_10_ and ECL_10_ values using constrained nonlinear regression^a^

	Segment	Sex	EC_05-flux_	ECL_05-flux_	Hill slope^b^	Max^b^	*R*^2^
			(EC_10-flux_)	(ECL_10-flux_)			
Diffuse hyperplasia	d, i	m, f	0.88	0.56	1.7	0.82	0.94
			(1.4)	(0.98)			
Adenoma	d, j, i	m, f	5.9	4.2	1.7	0.82	0.85
			(9.2)	(7.3)			
Carcinoma	d, j, i	m, f	15.1	9.4	1.7	0.82	0.57
			(23.5)	(14.5)			

d, duodenum; f, female; i, ileum; j, jejunum; m, male.

a The minimum parameter was constrained to be zero; the maximum parameter was constrained to be between 0 and 1 (and shared); the Hill slope parameter was constrained to be shared.

b Values are global values.

##### Interspecies extrapolation

Internal doses corresponding to the PODs for small intestinal hyperplasia and tumors in mice can be extrapolated to humans using the human PBPK model developed for Cr (Kirman *et al.*, 2013). However, limitations in the available human data, specifically the lack of data regarding the relative uptake of chromium in the human duodenum, jejunum and ileum, preclude the use section-specific flux estimates for interspecies extrapolation. Therefore, two measures of total Cr(VI) flux were identified as internal dose surrogates to be used to extrapolate from mice to humans: (1) the flux of Cr(VI) leaving the stomach lumen (normalized to per liter of SI tissue per day (mg Cr(VI) l^–1^ day^–1^) (which we term “pyloric flux” for simplicity), and (2) the flux of Cr(VI) entering the total SI tissue (normalized to per kg SI tissue per day (mg Cr(VI) l^–1^ day^–1^) (which we term “intestinal flux” for simplicity). In the mouse, the latter estimate is calculated as the segment mass-weighted average for Cr(VI) flux in total SI. Both measures of Cr(VI) flux were related to total SI tissue response (i.e., including the duodenum, jejunum and ileum together) in the mouse using an assumption of dose additivity (i.e., total SI response is predicted using the sum of the section flux estimates). In this way, for example, a BMD_05_ value corresponds to the Cr(VI) internal dose that produces a 5% response rate in the total SI, which is composed of relative section contributions of approximately 4.4% response in mouse duodenum, 0.55% response in mouse jejunum and 0.05% response in mouse ileum. The pyloric flux surrogate [flux of Cr(VI) leaving the stomach lumen] can be predicted with a reasonable degree of certainty in humans, because it depends primarily on our understanding of gastric transit rates obtained from the published literature (ICRP, 2002) and the reduction of Cr(VI) in human gastric contents that were measured *ex vivo* (Kirman *et al.*, 2013). Use of the pyloric flux surrogate for interspecies extrapolation assumes that the toxicokinetic processes for Cr(VI) in SI lumen and tissue are qualitatively and quantitatively similar for mice and humans. The intestinal flux surrogate [flux of Cr(VI) entering the total SI] can also be predicted with a reasonable degree of certainty, because it depends on available human toxicokinetic data (Kirman *et al.*, 2013). The key assumptions for this flux surrogate are (1) that the SI serves as the primary site of Cr absorption, and therefore, measurements of Cr in human plasma and urine (obtained from numerous Cr pharmacokinetic studies published in the literature) predominantly reflect Cr that had been absorbed in the SI (but cannot differentiate absorption via each intestinal segment), and (2) that the toxicokinetic processes for Cr(VI) when it reaches small intestinal tissue are qualitatively and quantitatively similar for mice and humans. All three estimates of Cr(VI) flux used in this assessment are depicted graphically in Appendix A (see Fig. A.2).

Human equivalent lifetime average daily doses (LADD_HE_) that correspond to the mouse internal POD values were calculated using the human PBPK model by considering variation in toxicokinetic processes for Cr(VI) as a function of age using the following five age groups: (1) neonate (0–3 months); (2) infant/child (0.25–6 years); (3) youth (6–18 years); (4) adult (18–60 years); and (5) elderly (60–75 years). Human exposures via drinking water were considered to be of primary importance for Cr(VI), and therefore, age group-specific exposure scenarios were developed based on the drinking water consumption pattern data (Barraj *et al.*, 2009). For the purposes of modeling, the average number of drinking water events per day for each age group from this study was rounded up to the next-highest even number, with half of the exposure events assumed to occur on an empty stomach (i.e., fasted state between meals), and the other half of the exposure events assumed to occur in a fed state (e.g., water consumed with meals). Exposure events (four to six per day) were defined to occur over 1 h intervals, based on the hourly consumption pattern data (Barraj *et al.*, 2009). In addition to exposure-event scheduling, several gastrointestinal parameters were modeled to vary over the course of a day, including gastric pH, gastric transit half-time and gastric reducing equivalents. Details on the application of the human PBPK model for chromium to risk assessment are summarized in Appendix B. Human equivalent LADDs corresponding to the mouse POD values for small intestinal hyperplasia and tumors were calculated as the time-weighted average for each age group, based on the two Cr(VI) flux surrogates (pyloric flux and total intestinal flux).

#### Chronic oral reference dose derivation

A range of LADD_HE_ values were calculated for diffuse hyperplasia and tumor formation based on two modeling approaches (BMD modeling and CNR) and two human dose surrogates (pyloric flux and total intestinal flux) (Appendix B). Because species differences in pharmacokinetics were accounted for by using rodent and human PBPK models, the BMDL and ECL values were each reduced threefold to account for potential remaining uncertainties in pharmacodynamics when extrapolating from mice to humans. The human PBPK model was then used to estimate external doses to humans that result in these two internal dose metrics for each outcome of interest (i.e., hyperplasia, adenomas and carcinomas). Values based on BMDL_05_ and ECL_05_ are shown in Table 6. Values based on a 10% response can be found in Appendix B.

**Table 6:** Human LADD values corresponding to mouse POD values

		Internal dose (mg Cr(VI) kg^–1^ SI day^–1^)			External dose (mg Cr(VI) kg^–1^ BW day^–1^)	
		Mice	Human		Human LADD^b^	
Response	POD	SI sectional flux	Pyloric flux^a^	Total SI flux^a^	Pyloric flux	Total SI flux
Hyperplasia	BMDL_05_	0.84	0.75	0.092	0.061	0.059
	ECL_05_	0.56	0.49	0.061	0.041	0.040
Adenoma	BMDL_05_	4.5	4.1	0.49	0.20	0.18
	ECL_05_	4.2	3.8	0.46	0.19	0.17
Carcinoma	BMDL_05_	16	15	1.8	0.44	0.37
	ECL_05_	9.4	8.6	1.0	0.31	0.27

BMDL, benchmark dose values and their 95% lower confidence limits; ECL, effective concentration values and their 95% lower confidence limits; LADD, lifetime average daily doses; POD, points of departure; SI, small intestine.

a This value has already been divided by a threefold UF_A_ (see text and Appendices A and B).

b The LADD is a time-weighted average for five age groups (see Appendix B).

The multiple dose–response approaches described herein support a conclusion that diffuse hyperplasia is a more sensitive endpoint than tumor formation. Moreover, the MOA for Cr(VI)-induced intestinal tumors suggests that protection against the precursor effect of diffuse hyperplasia will also be protective of intestinal neoplasms. Therefore, only LADD_HE_ values for diffuse hyperplasia were considered for RfD derivation. The LADD_HE_ values for diffuse hyperplasia based on BMD modeling and CNR ranged from 0.04 to 0.06 mg kg^–1^ bodyweight day^–1^ (Table 6). Because the BMD methodology is recommended by US EPA for dose–response modeling, only the mean LADD_HE_ values based on the BMDL_05_ values were considered for RfD derivation at this time. This mean LADD_HE_ value was reduced by a 10-fold intraspecies uncertainty factor (UF_H_) to account for human variability in Cr(VI) disposition and pharmacodynamic responses. A database uncertainty factor (UF_D_) was deemed unnecessary due to the availability of reproductive and developmental toxicity studies in multiple species; adverse effects from these studies were less sensitive than those in the gastrointestinal tract (US EPA, 2010). The resulting chronic RfD value is 0.006 mg kg^–1^ day^–1^, which is considered protective of the noncancer and cancer effects of Cr(VI) in the SI (Table 7).

**Table 7:** Oral RfD and DWEL values for Cr(VI)

Endpoint	LADD_HE_ (mg kg^–1^ day^–1^)	UF_H_	RfD (mg kg^–1^ day^–1^)	DWEL (µg l^–1^)
Diffuse hyperplasia	0.06^a^	10^b^	0.006	210^c^

DWEL, drinking water equivalent level; LADD, lifetime average daily doses; RfD, reference dose.

a Mean BMDL_05_ from Table 6 (a threefold UF_A_ is already incorporated).

b See text for discussion.

c DWEL = RfD mg kg^–1^ day^–1^ × 70 kg ÷ 2 l

### Discussion

A series of recent studies into the MOA of Cr(VI) in the small intestine indicate that the weight of evidence supports a nonmutagenic MOA based on chronic intestinal wounding leading to compensatory regenerative crypt hyperplasia and, ultimately, intestinal carcinogenesis (Thompson *et al.*, 2013). These findings establish that the MOA for Cr(VI)-induced intestinal tumors is not linear in the low-dose region. Concentrations of Cr(VI) that do not induce cytotoxicity and regenerative crypt proliferation are unlikely to increase the risk of intestinal cancer (see Fig. 3). For carcinogens that induce cancer through such nonlinear mechanisms, the US EPA has recommended development of RfD values (US EPA, 2005). An RfD is defined by the US EPA as “an estimate (with uncertainty spanning perhaps an order of magnitude) of a daily exposure to the human population (including sensitive subgroups) that is likely to be without an appreciable risk of deleterious effects during a lifetime.” Moreover, it is said to “provide quantitative information for use in risk assessments for health effects known or assumed to be produced through a nonlinear (possibly threshold) mode of action” (http://www.epa.gov/iris/help_ques.htm*)*. In this regard, RfD values based on intestinal irritation induced by captan and folpet have been deemed protective of intestinal cancer (Gordon, 2007; US EPA, 2004).

The RfD developed herein is derived from a very rich data set. By using a rodent PBPK model to estimate target tissue doses achieved in multiple intestinal segments of all treated animals (male and female) in the 2-year bioassay, incidence values at multiple dose levels could be used to create a robust dose–response curve. Examining each intestinal segment within the proper context of tissue dose, dose–response data for the segment achieving the lowest internal dose (i.e., ileum) can be used to improve our understanding of the potential low-dose risks associated with the high internal doses achieved in upper segments of the intestine (i.e., duodenum and jejunum). Using this robust data set, standard BMD modeling was used to calculate BMDL values for diffuse hyperplasia based on internal dose (i.e., SI section flux). In addition, CNR was employed to develop ECL values for the same endpoint. Although CNR is often used to share parameters for the same endpoint (e.g., receptor activation by two congeners), it could be used, in theory, to share parameters between two related phenomena when plotted on the same axes (i.e., dose vs. incidence). Notably, the POD values using BMD and CNR modeling were remarkably similar. The range of POD estimates for each endpoint were quite narrow (i.e., < 2-fold; Table 6). Obtaining similar findings using multiple modeling approaches strengthens the confidence in the results. Moreover, these POD values for hyperplasia, adenomas and carcinomas are consistent with the progression of intestinal cancer (Grady and Carethers, 2008; Greaves, 2012). To our knowledge, this is the first example of using CNR to share parameters to characterize the progression of disease (e.g., hyperplasia to adenoma to carcinoma); additional case examples are needed to assess the general applicability of this approach in risk assessment.

The proposed RfD (0.006 mg kg^–1^ day^–1^) is less than 10-fold higher than the RfD previously derived by US EPA (2010). In their draft assessment, US EPA's BMD modeling of diffuse hyperplasia based on applied dose (mg kg^–1^ bodyweight) in female mice resulted in an RfD of 0.0009 mg kg^–1^ day^–1^ [0.09 mg kg^–1^ (the BMDL_10_ for diffuse hyperplasia) divided by 10-fold uncertainty factors for UF_A_ and UF_H_, each]. A major difference between these RfD values is the treatment of the critical effect. US EPA analyzed diffuse hyperplasia in males and females separately, despite evidence that this effect was similar in both sexes (Figs 1 and 3). When modeling diffuse hyperplasia in this manner based on applied dose, acceptable BMD modeling fits could only be achieved by dropping the two highest dose groups from the analysis – leaving only two treatment doses and a control group for quantitative modeling (Table 2). In contrast, the modeling approach described herein uses an internal dose metric that allows for the derivation of PODs based on 13 data points normalized across intestinal segments (duodenum and ileum) for diffuse hyperplasia, and 24 data points for tumor formation (in duodenum, jejunum and ileum). Another difference in the RfD values proposed herein and those by US EPA is the application of uncertainty factors. US EPA applied 10-fold default values each for UF_A_ and UF_H_ (US EPA, 2010). The newly developed PBPK models allows for a reduction in the UF_A_ to threefold due to accounting for species differences in the disposition of Cr(VI). In addition, the human PBPK model allows for development of an RfD based on a LADD, which includes life-stage-specific adjustments to pharmacokinetic aspects of Cr(VI) disposition [e.g., stomach pH variability, which affects the rates of Cr(VI) reduction throughout life] and thus provides a more scientifically robust quantitative description of dose. Nevertheless, we conservatively included a full 10-fold UF_H_ value to account for interindividual human variability.

The use of the rodent PBPK model to convert the applied doses in the animal study to an internal tissue dose metric, and the human PBPK model to convert the PODs to HEDs, offers a vast improvement over using the applied study doses for deriving RfDs. Some sources of uncertainty remain in the PBPK models for chromium in mice and humans, many of which have been discussed previously (Kirman *et al.*, 2013; Kirman *et al.*, 2012). With respect to the human PBPK model, the data available for chromium in exposed humans are limited to plasma, erythrocytes and urine (Kirman *et al.*, 2013), and for this reason, the Cr(VI) flux estimates into the total SI from the human model are uncertain. To address this limitation, a second flux estimate [Cr(VI) leaving the stomach], which can be estimated with a greater degree of certainty as it depends on parameters that are relatively well characterized (human stomach transit times and human gastric reduction rates), was included in the assessment. The two Cr(VI) flux estimates evaluated (pyloric and intestinal flux) have separate bases and assumptions, but nevertheless result in nearly identical estimates of risk, differing by less than a factor of 2. Hence, this source of uncertainty is relatively small.

One of the largest sources of uncertainty relates to the relative timing of Cr(VI) exposure events and normal diurnal variation in gastrointestinal parameters such as pH, reducing equivalents and gastric transit due to the presence or absence of food in the stomach. For the human equivalent doses presented above, an assumption was made that 50% of the drinking water exposure events per day occur during a fed state and 50% during a fasted state. Because some factors favor greater gastric reduction during the fed state (e.g., higher reducing equivalent concentrations, longer gastric transit half-life), while other factors favor greater gastric reduction during a fasted state (e.g., lower pH resulting in a higher rate of reduction), it is not obvious which state results in greater delivery of Cr(VI) to the SI. Model predictions suggest that assuming 100% of exposure events during a fasted state will result in slightly larger estimates of daily internal dose to the SI than estimated in this assessment (by a factor of approximately 2–5), while assuming 100% of exposure events during a fed state will result in slightly lower estimates of internal dose to the SI than estimated in this assessment (by a factor of approximately 20–50%) (Appendix B). However, neither of these extreme assumptions is likely to remain constant over a lifetime.

The rate of Cr(VI) reduction in human stomach fluid in the fed state in the human PBPK model is based on samples from fasted individuals at pH 5–7 because samples from fed individuals were not available for study of Cr(VI) reduction kinetics (Kirman *et al.*, 2013). It is known that the stomach pH increases immediately following a meal because the introduction of food dilutes acidic stomach fluid. Because we do not have data on the reduction rate in actual fed conditions, we have had to rely on reduction rate data for fasted individuals at a higher pH than normal fasting conditions, at which the pH is ∼ 1.5. As such, the current model does not allow us to account quantitatively for any differences in reduction rate that might be expected with the release of gastric acid and enzymes that occur with the consumption of food. We expect that the Cr(VI) reduction rate may be underestimated for a fed state in the PBPK model, resulting in an overestimation of the transfer of Cr(VI) to the SI in fed conditions.

Although the assessment presented here specifically included modeling of different age groups, to account for differences in toxicokinetic factors as a function of age, it did not explicitly consider other conditions or disease states that may affect risk. For example, individuals who take proton-pump inhibitors (PPIs) are expected to have higher gastric pH levels, and because of the pH dependence of Cr(VI) reduction, have comparatively lower rates of Cr(VI) reduction in the gastric lumen when taking these medications. In fact, model predictions suggest that daily Cr(VI) flux estimates may be three- to fourfold higher among PPI users, based upon the pH profile of Atanassoff *et al.* (1995), than in individuals with normal stomach conditions. However, PPI medication is recommended for relatively short durations and as a result, the LADD value for PPI users is nearly identical to that for normal individuals. For example, assuming that an adult uses PPIs for 30 months (intermittently over a lifetime) (Dharmarajan *et al.*, 2008) and exhibit daily gastric pH consistent with previous reports (Atanassoff *et al.*, 1995), the model predicts that the lifetime average daily dose increases by approximately 7–10%. Because the variability in LADD estimates with PPI usage and with varying assumptions regarding water consumption patterns is small, the 10-fold UF_H_ used to calculate the RfD is considered to be adequately protective of these known variables of human sensitivity. Importantly, the use of our human PBPK model allows for the evaluation of sensitive life stages and conditions that otherwise could not be assessed quantitatively, and therefore increases confidence in the RfD.

Finally, Cr(VI) is prevalent in some US drinking water supplies at low concentrations (∼1–5 µg l^–1^) (AWWA, 2004; CDPH, 2011), and therefore, it is of significant public health interest to understand the potential cancer hazard associated with these typical exposures. The chronic drinking water equivalent level calculated from the RfD derived herein (0.006 mg kg^–1^ day^–1^), and the application of standard assumptions regarding drinking water consumption (2 l day^–1^) for a 70 kg individual, results in a drinking water concentration of 210 µg l^–1^. This value is greater than the current federal MCL for total Cr of 100 µg l^–1^ and is well above levels of Cr(VI) in drinking water supplies. Thus, typical concentrations of Cr(VI) in the US drinking water supply are not expected to increase the risk of intestinal cancer, and the current federal MCL of 100 µg l^–1^ is protective against increased intestinal cancer risk.

#### Sponsors

The authors employment affiliations are as shown on the cover page. Both ToxStrategies and Summit Toxicology are private consulting firms providing services to private and public organizations on toxicology and risk assessment issues. The authors [CT, CK, DP, LH, SH, MH] have presented study findings in meetings with regulators including public meetings on behalf of the Cr(VI) Panel of the American Chemistry Council (ACC). DP has also been an expert in litigation involving Cr(VI), which was unrelated to this research or ACC.

## Supporting Information

Supporting info item
