# Supplementary material for: A chronic oral reference dose for hexavalent chromium-induced intestinal cancer
Source: J Appl Toxicol. 2013 Aug 14;34(5):525–36. doi: 10.1002/jat.2907 (PMC4282340; doi:10.1002/jat.2907)
Supplement: Supplementary file 1 — Supporting info item [file jat0034-0525-SD1.docx]

**Appendix A. Calculation of Lifetime Average Daily Doses of Chromium Delivered to Mouse Small Intestines in the NTP Bioassay Using PBPK Modeling**

**A.1 Introduction**

A physiologically based pharmacokinetic (PBPK) model has been developed for chromium in rodents following oral exposures (Kirman et al., 2012). Inspection of initial model fits to all time points reveal some difficulties in fitting some of the data from this bioassay (**Figure A.1**), including underestimating urinary excretion at early time points and overestimating plasma at late time points. These difficulties likely stem from: (1) changes in dose over time due to age-related changes in drinking water rate and body weight; and (2) age- and/or treatment-related changes in plasma clearance and urinary excretion. These possibilities were noted by EPA scientists during their review of the rodent PBPK model. Model fitting difficulties were not encountered with the toxicokinetic data from the chromium picolinate bioassay (Figure 9 of Kirman et al., 2012), possibly because the test chemical was administered in feed instead of drinking water.

Although the toxicokinetic data collected by NTP (2008) are for systemic tissues and urinary excretion, which are not directly related to the target tissue of interest (i.e., small intestines), because the target tissue also serves as the primary site of chromium absorption, the toxicokinetic data indicate the extent to which chromium has passed through the small intestines (i.e., Cr flux). To improve the model fits and therefore confidence in model predictions for the mass of chromium passing through the small intestines, reaching systemic tissues, and excreted in the urine, the following approach was adopted.

**A.2 Methods**

The toxicokinetic data collected for mice in the NTP bioassay were separated into two groups: (1) early time points (data collected during weeks 1 and 2 of the study); and (2) late time points (data collected during weeks 26 and 53 of the study). Age-, sex- and group-specific values for chromium dose, drinking water consumption rate, and body weight were calculated from information provided in the bioassay (NTP, 2008) and are summarized in **Table A.1**. The model was then fit to the early and late data sets separately, allowing for some parameter values to change as a result of either age or treatment. For tissues important to Cr(III) storage (liver, kidney, bone, lumped other tissues, erythrocytes), the predicted tissue concentrations at the end of the early time simulations were used as initial concentrations for the late time simulations. All PBPK modeling was performed using the Microsoft Excel Add-In for acslX (AEgis TG). Revised model parameters are provided in **Table A.2**.

For calculating the lifetime average daily dose (LADD), the mouse lifetime was split into two time periods: (1) early life, for week 1-14; and (2) late life, for weeks 15-106. A time point of 14 weeks was selected since it reflects the mid-point between the toxicokinetic data collection time points for weeks 2 and 26. PBPK model parameters estimated for early time points (**Table A.2**, column 2), along with dosing information for weeks 1-14 (**Table A.1**, columns 9-11) were used to estimate the daily average internal dose for weeks 1-14. Similarly, PBPK model parameters estimated for late time points (**Table A.2**, column 3), along with dosing information for weeks 15-106 (**Table A.1**, columns 12-14) were used to estimate the daily average internal dose for weeks 15-106. LADD values for the two time periods were calculated as time-weighted averages for five PBPK-derived measures of internal dose [i.e., estimates of Cr(VI) flux]:

1. *Duodenum Flux (amtabsd6)* – Reflects the amount of Cr(VI) taken up into duodenum, per kg duodenum per day.
2. *Jejunum Flux (amtabsj6)* – Reflects the amount of Cr(VI) taken up into jejunum, per kg jejunum per day.
3. *Ileum Flux (amtabsi6)* – Reflects the amount of Cr(VI) taken up into ileum, per kg ileum per day.
4. *Pyloric Flux (amt2d6)* – Reflects the amount of Cr(VI) released from the stomach lumen to the small intestines lumen, per kg small intestines per day.
5. *Small Intestines Flux (amtabs6)* – Reflects the amount of Cr(VI) taken up into the entire small intestines (duodenum, jejunum, ileum combined), per kg small intestines per day.

A depiction of all five dose measures is provided in **Figure A.2**. The first three dose measures (sectional flux estimates) were used in the risk assessment to characterize the dose-response relationship (i.e., BMD modeling), while the latter two dose measures were used to extrapolate results from mice to humans.

**A.3 Results**

Model fits to the toxicokinetics data are provided in **Figures A.3** and **A.4**, which indicate notable improvement in model fits, particularly for the plasma and urine data when compared to initial fits (**Figure A.1**). The percentage of model predictions that are within a factor of 3 of the measured data points increases from 71% for the initial model (**Figure A.1**) to 86% for the refined model (**Figures A.3** and **A.4**). LADD values expressed in terms of SI section flux estimates are provided in **Table A.3** for each treatment group in the NTP bioassay. An example conversion of the point of departure values expressed in terms of SI section flux (used for dose-response assessment) to pyloric and total SI flux (used for interspecies extrapolation) is provided in **Figure A.5**.

Point of departure values expressed in terms SI section, pyloric, and total SI flux estimates are provided in **Table A.4**. For the purposes of converting SI section flux estimates to total SI flux estimates, section flux estimates for each section were summed. In this way, a benchmark response rate (BMR) of 0.10 corresponds to individual response rates of approximate 0.089, 0.01, and 0.001 for the duodenum, jejunum, and ileum, respectively.

**A.4 References**

Kirman, C. R., Hays, S. M., Aylward, L. L., Suh, M., Harris, M. A., Thompson, C. M., Haws, L. C. and Proctor, D. M. (2012). Physiologically based pharmacokinetic model for rats and mice orally exposed to chromium. Chem Biol Interact 200, 45-64.

NTP (2008). National Toxicology Program technical report on the toxicology and carcinogenesis studies of sodium dichromate dihydrate (CAS No. 7789-12-0) in F344/N rats and B6C3F1 mice (drinking water studies), NTP TR 546. NIH Publication No. 08-5887.

**Table A.1. Age-, Sex-, and Group-Specific Model Parameters Calculated for the NTP Mouse Bioassay (NTP, 2008)**

|  |  | **Parameter Values for Fitting Data Sets** | | | | | | **Parameter Values for Calculating LADD** | | | | | |
| --- | --- | --- | --- | --- | --- | --- | --- | --- | --- | --- | --- | --- | --- |
|  |  | **Week 1-2** | | | **Weeks 3-53** | | | **Weeks 1-14** | | | **Weeks 15-106** | | |
| **Sex** | **Treatment Group (mg SDD/L)** | **Dose (mg Cr(VI)/kg-day)** | **Drinking Water Rate (L/hr/kg)** | **Body Weight (kg)** | **Dose (mg Cr(VI)/kg-day)** | **Drinking Water Rate (L/hr/kg)** | **Body Weight (kg)** | **Dose (mg Cr(VI)/kg-day)** | **Drinking Water Rate (L/hr/kg)** | **Body Weight (kg)** | **Dose (mg Cr(VI)/kg-day)** | **Drinking Water Rate (L/hr/kg)** | **Body Weight (kg)** |
| Female | 14.3 | 0.80 | 0.0067 | 0.018 | 0.46 | 0.0033 | 0.038 | 0.67 | 0.0054 | 0.024 | 0.24 | 0.0020 | 0.057 |
|  | 57.3 | 3.1 | 0.0064 | 0.018 | 1.8 | 0.0033 | 0.036 | 2.6 | 0.0053 | 0.024 | 0.94 | 0.0019 | 0.055 |
|  | 172 | 7.7 | 0.0053 | 0.018 | 4.8 | 0.0030 | 0.033 | 6.5 | 0.0044 | 0.023 | 2.7 | 0.0018 | 0.051 |
|  | 516 | 16.5 | 0.0038 | 0.018 | 12 | 0.0026 | 0.030 | 14.8 | 0.0034 | 0.022 | 7.9 | 0.0018 | 0.045 |
| Male | 14.3 | NA | | | | | | 0.58 | 0.0046 | 0.033 | 0.43 | 0.0036 | 0.052 |
|  | 28.6 |  |  |  |  |  |  | 1.2 | 0.0046 | 0.033 | 0.88 | 0.0037 | 0.052 |
|  | 85.7 |  |  |  |  |  |  | 3.3 | 0.0045 | 0.032 | 2.3 | 0.0032 | 0.052 |
|  | 257.4 |  |  |  |  |  |  | 7.9 | 0.0036 | 0.029 | 5.4 | 0.0025 | 0.049 |

NA = Not Applicable. Toxicokinetic data were collected in female mice only.

**Table A.2. Model Parameter Values for Mouse PBPK Simulations**

|  |  |  | **Time Period-Specific Average Value** | |
| --- | --- | --- | --- | --- |
| **Description** | **PBPK Model Parameter** | **Lifetime Average Value (as used in Kirman et al., 2012)** | **Week 1-2** | **Week 3-53** |
| Body weight | bw | Lifetime average | Time-Specific (Table A.1) | Time-Specific (Table A.1) |
| Transfer of Cr(III) from plasma to kidney | kinccr | 0.008 | 0.003 | 0.008 |
| Transfer of Cr(III) from plasma to kidney | kintcrk | 0.0002 | 0.00025 | 0.0002 |
| Saturable absorption term for Cr(VI) in the small intestines | kmabs6 | 3 | 2 | 3 |
| Transfer of Cr(III) from liver to plasma | koutccrl | 0.000016 | 0.00003 | 0.000016 |
| Transfer of Cr(VI) from plasma to erythrocytes | krbcin6 | 0.036 | 0.015 | 0.036 |
| Urinary excretion | kurcc | 0.00007 | 0.0002 | 0.00007 |
| Oral dose Cr(VI) | od6 | Lifetime average | Time-Specific (Table A.1) | Time-Specific (Table A.1) |
| Drinking water rate | rdrinkc | Lifetime average | Time-Specific (Table A.1) | Time-Specific (Table A.1) |
| Saturable absorption of Cr(VI) in the stomach | vmabs6s | 0.0025 | 0.0006 | 0.0025 |

**Table A.3. PBPK-Derived Lifetime Average Daily Dose Estimates***

|  | **Female Mice** | | | | **Male Mice** | | | |
| --- | --- | --- | --- | --- | --- | --- | --- | --- |
| **Weeks** | **Treatment Group (mg SDD/L)** | **Duodenal Flux (amtabsd6)** | **Jejunal Flux (amtabsj6)** | **Ileum Flux (amtabsi6)** | **Treatment Group (mg SDD/L)** | **Duodenal Flux (amtabsd6)** | **Jejunal Flux (amtabsj6)** | **Ileum Flux (amtabsi6)** |
| 1-14 | 14.3 | 1.13E+01 | 1.19E+00 | 1.43E-01 | 14.3 | 7.70E+00 | 8.37E-01 | 1.01E-01 |
|  | 57.3 | 2.94E+01 | 3.95E+00 | 5.23E-01 | 28.6 | 1.36E+01 | 1.63E+00 | 2.04E-01 |
|  | 172 | 4.47E+01 | 7.32E+00 | 1.09E+00 | 85.7 | 2.54E+01 | 3.68E+00 | 5.07E-01 |
|  | 516 | 5.53E+01 | 1.00E+01 | 1.58E+00 | 257.4 | 3.76E+01 | 6.41E+00 | 9.72E-01 |
| 15-106 | 14.3 | 1.61E+00 | 1.78E-01 | 2.17E-02 | 14.3 | 2.93E+00 | 3.21E-01 | 3.87E-02 |
|  | 57.3 | 5.54E+00 | 6.71E-01 | 8.53E-02 | 28.6 | 5.42E+00 | 6.28E-01 | 7.77E-02 |
|  | 172 | 1.24E+01 | 1.74E+00 | 2.39E-01 | 85.7 | 1.09E+01 | 1.46E+00 | 1.94E-01 |
|  | 516 | 2.22E+01 | 3.75E+00 | 5.68E-01 | 257.4 | 1.79E+01 | 2.82E+00 | 4.11E-01 |
| 1-106 | 14.3 | 2.88E+00 | 3.12E-01 | 3.77E-02 | 14.3 | 3.56E+00 | 3.89E-01 | 4.69E-02 |
|  | 57.3 | 8.69E+00 | 1.10E+00 | 1.43E-01 | 28.6 | 6.50E+00 | 7.60E-01 | 9.43E-02 |
|  | 172 | 1.66E+01 | 2.48E+00 | 3.51E-01 | 85.7 | 1.28E+01 | 1.75E+00 | 2.36E-01 |
|  | 516 | 2.66E+01 | 4.58E+00 | 7.01E-01 | 257.4 | 2.05E+01 | 3.29E+00 | 4.85E-01 |

*All internal flux estimates are expressed in terms of mg Cr(VI)/kg tissue (SI section or total) per day.

**Table A.4. Points of Departure for Mouse SI Endpoints Expressed in terms of Internal Dose**

|  |  |  | **BMDL (mg Cr(VI)/kg SI-day)** | | |
| --- | --- | --- | --- | --- | --- |
| **Dose-Response Modeling (Software)** | **Response** | **BMR** | **SI Section Flux*** | **Pyloric Flux** | **Total SI Flux** |
| Benchmark Dose (BMDS) | Hyperplasia | 0.10 | 1.4 | 3.8 | 0.46 |
|  |  | 0.05 | 0.84 | 2.2 | 0.28 |
|  | Adenoma | 0.10 | 8.3 | 23 | 2.7 |
|  |  | 0.05 | 4.5 | 12 | 1.5 |
|  | Carcinoma | 0.10 | 22 | 61 | 7.2 |
|  |  | 0.05 | 16 | 45 | 5.4 |
| Constrained Nonlinear Regression (Graphpad Prism) | Hyperplasia | 0.10 | 0.98 | 2.6 | 0.32 |
|  |  | 0.05 | 0.56 | 1.5 | 0.18 |
|  | Adenoma | 0.10 | 7.3 | 20 | 2.4 |
|  |  | 0.05 | 4.2 | 11 | 1.4 |
|  | Carcinoma | 0.10 | 15 | 40 | 4.8 |
|  |  | 0.05 | 9.4 | 26 | 3.1 |

*For the purposes of converting flux estimates, section flux estimates for each section were summed. In this way, a BMR of 0.10 corresponds to individual response rates of approximate 0.089, 0.01, and 0.001 for the duodenum, jejunum, and ileum, respectively.

Figure A.1 Initial PBPK Model Fits to All Time Points for Mouse Toxicokinetics Data (NTP, 2008).

Figure A.2. Depiction of Flux Values Used In the Cr(VI) Risk Assessment for Mouse SI Effects

Figure A.3 Refined PBPK Model Fits to Early Time Points for Mouse Toxicokinetics Data (NTP, 2008).

Figure A.4 Refined PBPK Model Fits to LateTime Points for Mouse Toxicokinetics Data (NTP, 2008).

Fig. A.5 Example Conversion of Point of Departure from Mouse Sectional Flux Estimates to Pyloric and Total Flux Estimates. See first row of Table A.4.

**Appendix B. Using the PBPK Model for Cr(VI) to Calculate Human Equivalent Lifetime Average Daily Doses**

**B.1 Introduction**

A physiologically based pharmacokinetic (PBPK) model has been developed to describe the toxicokinetics of hexavalent chromium [Cr(VI)] in humans (Kirman et al., 2013). The PBPK model was used to support a human health risk assessment (HHRA) based upon mouse small intestinal (SI) tumors (NTP, 2008). The methods and results for human model application are summarized below.

**B.2 Methods**

Key factors contributing to the delivery of Cr(VI) to the small intestines (SI) were identified by sensitivity analyses (Kirman et al., 2012, 2013), and include gastric pH, Cr(VI) reduction rate constant, gastric transit time, and gastric reducing equivalents. These factors affect the rate of Cr(VI) reduction in gastric contents, with a higher delivery of Cr(VI) resulting from higher pH values (which causes slower rates of Cr(VI) reduction), shorter stomach transit times, and lower reducing equivalent concentrations. The PBPK model was used to address several important sources of variation: (1) variation across age groups; (2) diurnal variation/exposure timing; and (3) sensitive subpopulations. Individual variation was not addressed in this assessment, but is expected to be part of future work.

**B.2.1 Assessment of Variation Across Age Groups**

Age-specific differences in the key factors were incorporated in the modeling to estimate human equivalent lifetime average daily dose corresponding to point of departures determined for mouse SI lesions. Consideration of age-specific parameters in the assessment was considered important since some age groups (e.g., neonates) normally exhibit baseline peak and/or peak pH values than adults (Dressman et al., 1990; Russell et al., 1993; Nagita et al., 1996; Omari and Davidson, 2003). Elevated baseline gastric pH in neonates is relatively short-lived, show a significant drop during the 2^nd^ week post-partum (Sondheimer et al., 1985), and reaches approximate adult levels by 3 months of age (Milsap et al., 1994). Because of the differences in gastric pH, differences in the pH-dependent reduction of Cr(VI) in the GI lumen are expected between age groups. The PBPK model was used to simulate exposures to 5 different age groups, including neonates (0-3 months), infants/children (3 months-6 years), youths (6-18 years), adults (18-60 years), and elderly (60-75 years). Age-specific values for body weight, tissue and lumen volumes, and transit rates were obtained from published reviews (ICRP, 2002; USEPA, 2011), and are summarized in **Table B.1**.

**B.2.2 Assessment of Diurnal Variation and Exposure Timing**

The PBPK model was used to account for normal diurnal variation in gastric lumen factors. For example, in normal individuals, baseline gastric pH is typically between 1-3 between meals, but rises rapidly to levels of 5-7 at the start of a meal, then returning to baseline levels within a 2-4 hours (Dressman et al., 1990; Russell et al., 1993; Nagita et al., 1996; Omari and Davidson, 2003). The return of gastric pH from peak values during the meal to baseline exhibits different behavior for different age groups. In older adults and in infants, the return to baseline exhibits a linear behavior (Omari and Davidson, 2003; Dressman et al., 1990; Russell et al., 1993), while in young adults the return to baseline appears nonlinear (**Figure B.1**). For the risk assessment, a linear return to baseline gastric pH was assumed for neonates, infant/child, and elderly scenarios, and a nonlinear return to baseline was assumed for youth and adult scenarios. Dichotomous values for fed and fasted states were adopted for gastric transit rate and reducing equivalent concentration based on information from the published literature (ICRP, 2002; De Flora et al., 1987) and this study, where fed and fasted states are defined in terms of the presence or absence of food in the stomach lumen, respectively. 24-Hour time-profiles were developed for the following model parameters (model code parameter indicated in parentheses): gastric pH (phs), gastric transit rate (klsd), and concentration of reducing equivalents in gastric contents (cre0) (**Figure B.2**).

Because the delivery of Cr(VI) to the small intestines will be different when exposure occurs during a fed vs. during a fasted state, the PBPK model was used to assess the impact of the timing of Cr(VI) exposure events. For this assessment, half of the daily exposure events assumed to occur during a fasted state, and the other half of the daily exposure events assumed to occur during a fed state. The impact of assuming 100% of exposure events occurring during either state was also assessment. Exposures to Cr(VI) were assumed to occur via drinking water, with the number of exposure events/day estimated from Barraj et al. (2009). Specifically, the average number of daily drinking water events for each age group was rounded up to the next even number, with half of the events assumed to occur during a fasted state, and the other half assumed to occur during a fed state.

**B.2.3 Assessment of Sensitive Subpopulations**

The model was used to assess risk to specific populations (proton-pump inhibitor or PPI users) that may be sensitive to Cr(VI) due to elevation in gastric pH, which reduces the pH-dependent reduction of Cr(VI) in the GI lumen, which in turn results in greater delivery of Cr(VI) to the small intestines. For PPI user simulations, a 24-hour gastric pH profile was developed based on the data collected by Atanassoff et al. (1995). Specifically, the arithmetic mean for 4 treatment scenarios (2 medications, omeprazole and ranitidine, each at 2 dose levels) was used, and is depicted in **Figure B.1**. A duration of PPI use of 30 months was assumed in estimating the impact to LADD values, based on a reported mean duration of 18.2 months (SD = 16.6 months) for adults (Dharmarajan et al., 2008). LADD values were re-calculated by replacing 30 months of exposure as a normal adult with 30-months of exposure as a PPI user. This is considered to be a conservative evaluation since in the U.S., the FDA advises that no more than three 14-day treatment courses of PPIs should be used in one year (FDA, 2010).

**B.2.4 Calculation of Lifetime Average Daily Dose (LADD) for Mouse PODs**

Two estimates on internal dose were used to calculate human equivalent doses for points of departure determined for mouse SI effects: (1) Pyloric flux (amt2d6), which reflects the amount of Cr(VI) released from the stomach lumen to the small intestines lumen, per kg small intestines per day; and (2) Small intestines flux (amtabs6), which reflects the amount of Cr(VI) taken up into the entire small intestines, per kg small intestines per day. Human LADD estimates for Cr(VI) were determined using the following steps:

1. The relationship between administered dose of Cr(VI) and the two internal dose measures was estimated for each age group across a broad range of administered doses of Cr(VI) (0.0001-10 mg/kg).
2. At each administered dose level, the lifetime average internal dose was calculated as a time-weighted average of the internal dose estimated for each age group (**Figure B.3**).
3. The relationship between administered lifetime average daily dose (LADD) and lifetime average internal dose was characterized for both dose measures (**Figure B.4**). This relationship is linear at low doses (below 0.1 mg Cr(VI)/kg BW-day), but becomes nonlinear as GI reducing equivalents are predicted to become depleted.
4. Human equivalent LADD values corresponding to the mouse point of departure values were calculated by interpolating from the internal-external dose curves generated in Step 3.

All PBPK modeling simulations were performed using acslX and its add-in for Microsoft Excel (AEgis TG). Minor modifications were made to the PBPK model code to allow GI parameters to change over the course of a day (using acslX Table functions, see modified code in **Section B.4**).

**B.3 Results**

**B.3.1 Age-Group Contributions to LADD**

Age-group-specific contributions to lifetime average internal dose estimates are depicted in **Figure B.3**. The relative contributions of the dose received during each age period on LADD estimates for the two internal dose measure are as follows:

- Pyloric Flux (amt2d6): neonate (1.3%), infant/child (7.1%), youth (19%), adult (59%), and elderly (14%)
- Small Intestines Flux (amtabs6): neonate (2.6%), infant/child (9.1%), youth (20%), adult (51%), and elderly (17%)

As expected, exposure while adult (comprising 42 years or 56% of a 75-year lifetime) is the largest contributors to lifetime average estimates for internal dose.

**B.3.2 Diurnal Variation & Exposure Timing**

Example 24-hour time-profiles for the two internal doses estimated for each age group are depicted in **Figure B.2**. Based upon these profiles, exposure events that occur during a fasted state result in greater delivery to the small intestines than exposure events that occur during a fed state. This conclusion is not obvious since there are competing factors that contribute to overall delivery. During a fasted state, values for gastric transit rate (faster) and gastric reducing equivalents (lower) favor greater delivery of Cr(VI) to the small intestines, however the gastric pH during a fasted state (lower) favors lower delivery of Cr(VI).

An assessment of several alternative Cr(VI) exposure assumptions is provided below.

- *What if all Cr(VI) exposure events occur during a fasted state?* – When all of the daily Cr(VI) exposure were assumed to occur during a fasted state, the resulting doses delivered to the small intestines were 1.4- to 4.7-fold higher than estimated in this assessment.
- *What if 100% of Cr(VI) exposure occurs during a fed state?* - When all of the daily Cr(VI) exposure were assumed to occur during a fed state, the resulting doses delivered to the small intestines were 20-50% lower than estimated in this assessment.
- *What if all Cr(VI) exposure occurs during a single exposure event?* – When all of the daily Cr(VI) exposure were assumed to occur during a single exposure event, the resulting doses delivered to the small intestines were up to 8-fold higher than estimated in this assessment.

**B.3.3 Sensitive Subpopulations**

Flux estimates in PPI users are approximately 3- to 4-fold higher than normal adults. However, the impact of adult PPI use for 30 months (3 months/year for 10 years) on LADD estimates is relatively small. For calculations based on pyloric flux, adult PPI use increased the LADD values by up to 7%. Similarly, for calculations based on SI flux, adult PPI use increased the LADD values by up to 10%.

**B.3.4 Calculation of LADD Values for Mouse POD Values**

Based on curves predicted by the PBPK model for lifetime average internal doses as a function of administered LADD, LADD values were determined for the POD/UFa determined for mouse SI endpoints. An example calculation is provided in **Figure B.4**. Similar calculations were performed for the remaining POD values, and corresponding LADD values are provided in **Table B.2**.

**B.4 References**

Atanassoff, P. G., Brull, S. J., Weiss, B. M., Landefeld, K., Alon, E. and Rohling, R. (1995). The time course of gastric pH changes induced by omeprazole and ranitidine: a 24-hour dose-response study. Anesth Analg 80, 975-9.

Barraj, L., Scrafford, C., Lantz, J., Daniels, C. and Mihlan, G. (2009). Within-day drinking water consumption patterns: results from a drinking water consumption survey. J Expo Sci Environ Epidemiol 19, 382-95.

De Flora, S., Badolati, G. S., Serra, D., Picciotto, A., Magnolia, M. R. and Savarino, V. (1987). Circadian reduction of chromium in the gastric environment. Mutat Res 192, 169-74.

Dharmarajan TS, Kanagala MR, Murakonda P, Lebelt AS, Norkus EP. (2008). Do acid-lowering agents affect vitamin B12 status in older adults? J Am Med Dir Assoc. 9(3):162-7.

Dressman, J. B., Berardi, R. R., Dermentzoglou, L. C., Russell, T. L., Schmaltz, S. P., Barnett, J. L. and Jarvenpaa, K. M. (1990). Upper gastrointestinal (GI) pH in young, healthy men and women. Pharmaceutical research 7, 756-61.

U.S. FDA.2010. Possible increased risk of bone fractures with certain antacid drugs. http://www.fda.gov/ForConsumers/ConsumerUpdates/ucm213240.htm

ICRP (2002). Basic Anatomical and Physiological Data for Use in Radiological Protection: Reference Values. ICRP Publication 89.

Kirman, C. R., Aylward, L. L., Suh, M., Harris, M. A., Thompson, C. M., Haws, L. C., Proctor, D. M., Parker, W., Lin, S. and Hays, S. M. (2013). Physiologically Based Pharmacokinetic Model for Humans Orally Exposed to Chromium. Chemico-Biological Interations. Epub ahead of print.

Kirman, C. R., Hays, S. M., Aylward, L. L., Suh, M., Harris, M. A., Thompson, C. M., Haws, L. C. and Proctor, D. M. (2012). Physiologically based pharmacokinetic model for rats and mice orally exposed to chromium. Chem Biol Interact 200, 45-64.

Milsap RL, Jusko WJ. (1994). Pharmacokinetics in the infant. Environ Health Perspect. Dec;102 Suppl 11:107-10.

Nagita, A., Amemoto, K., Yoden, A., Aoki, S., Sakaguchi, M., Ashida, K. and Mino, M. (1996). Diurnal variation in intragastric pH in children with and without peptic ulcers. Pediatric research 40, 528-32.

NTP (2008a). National Toxicology Program technical report on the toxicology and carcinogenesis studies of sodium dichromate dihydrate (CAS No. 7789-12-0) in F344/N rats and B6C3F1 mice (drinking water studies), NTP TR 546. NIH Publication No. 08-5887.

Omari TI, Davidson GP. (2003). Multipoint measurement of intragastric pH in healthy preterm infants. Arch Dis Child Fetal Neonatal Ed. 2003 Nov;88(6):F517-20.

Russell, T. L., Berardi, R. R., Barnett, J. L., Dermentzoglou, L. C., Jarvenpaa, K. M., Schmaltz, S. P. and Dressman, J. B. (1993). Upper gastrointestinal pH in seventy-nine healthy, elderly, North American men and women. Pharm Res 10, 187-96.

Sondheimer JM, Clark DA, Gervaise EP. (1985). Continuous gastric pH measurement in young and older healthy preterm infants receiving formula and clear liquid feedings. J Pediatr Gastroenterol Nutr. 4(3):352-5.

U.S. EPA (2011a). Exposure Factors Handbook: 2011 Edition. Risk Assessment Forum EPA/600/R-09/052F.

**Table B.1. Age-Specific Model Parameters**

| **Parameter** | **Abbrev.** | **Neonate** | **Infant/Child** | **Youth** | **Adult** | **Elderly** | **Reference** |
| --- | --- | --- | --- | --- | --- | --- | --- |
| Age | -- | 0-0.25 yr | 0.25-6 yr | 6-18 yr | 18-60 yr | 60-75 yr | Professional judgement |
| Exposure Duration (years) | ED | 0.25 | 5.73 | 12 | 42 | 15 | Professional judgement |
| Body Weight (kg) | Wbody | 5.5 | 15.2 | 48.9 | 80 | 80 | EPA (2011) |
| Cr Exposure Events per Day | -- | 4 (2 fed; 2 fasted) | 4 (2 fed; 2 fasted) | 6 (3 fed; 3 fasted) | 6 (3 fed; 3 fasted) | 6 (3 fed; 3 fasted) | Barraj et al. (2009; split assumed) |
| Number of Meals/Day | -- | 6 | 4 | 3 | 3 | 3 | Professional judgement |
| Drinking Water Rate (L/kg-hr) | Rdrink | 0.0028 | 0.00088 | 0.00041 | 0.00054 | 0.00054 | ICRP (2002), USEPA (2011) |
| Food Intake Rate (kg/kg-hr) | Rfood | 0.0038 | 0.0039 | 0.0015 | 0.0012 | 0.0012 |  |
| Saliva Production Rate (L/kg-hr) | Rsal | 0.00063 | 0.00063 | 0.00063 | 0.00063 | 0.00063 | Values for adults is based on ICRP(2002) and USEPA(2011), which was assumed for all other age groups |
| Gastric Fluid Production Rate (L/kg-hr) | Rgif | 0.00104 | 0.00104 | 0.00104 | 0.00104 | 0.00104 | Values for adults is based on ICRP(2002) and USEPA(2011), which was assumed for all other age groups |
| Peak Gastric Lumen pH (fed) | PHPeak | 7 | 7 | 6 | 5 | 5 | Dressman et al. (1990); Russell et al. (1993); Nagita et al. (1996); Omari and Davidson 2003 |
| Baseline Gastric Lumen pH (fasted) | PHBase | 3.5 | 2 | 1.5 | 1.7 | 1.3 |  |
| Time to return to baseline gastric pH from peak (hr) | -- | 2 | 2 | 3 | 3 | 4 |  |
| Gastric pH return to baseline behavior | -- | Linear | Linear | Nonlinear | Nonlinear | Linear |  |
| pH-Dependent 2^nd^ Order Reduction Rate Constant | kredgifc | 44.5 | 44.5 | 44.5 | 44.5 | 44.5 | Adult value is based on model fits to data provided in **Table B.3**, which was assumed for all other age groups |
| Gastric Lumen Reducing Equivalents, fed (mg/L) | CRE0Fed | 30 | 30 | 30 | 30 | 30 | Adult value is based on DeFlora et al. (1987), which was assumed for all other age groups |
| Gastric Lumen Reducing Equivalents, fasted state (mg/L) | CRE0Fast | 7 | 7 | 7 | 7 | 7 | Adult value is based on model fits to data provided in **Table B.3**, which was assumed for all other age groups |
| Gastric Lumen Transit, Fed (hours) | KLSDFed | 1.25 | 1.17 | 1.17 | 1.38 | 1.38 | ICRP (2002) |
| Gastric Lumen Transit, Fasted (hours) | KLSDFast | 0.17 | 0.50 | 0.50 | 0.50 | 0.50 |  |
| Small Intestines Lumen Transit (hours) | KLIL | 4 | 4 | 4 | 4 | 4 |  |
| Small Intestines Lumen pH | PHSI | 6.5 | 6.5 | 6.5 | 6.5 | 7 | Kararli (1995); Russell et al. (1990) |
| SI Length (cm) | SIL | 120 | 150 | 220 | 270 | 270 | ICRP (2002) |
| SI Mass (fbw) | SIc | 0.015 | 0.014 | 0.0076 | 0.0078 | 0.0078 |  |
| SI Lumen Mass (fbw) | SILc | 0.010 | 0.0069 | 0.0045 | 0.0039 | 0.0039 |  |
| Stom Mass (fbw) | Sc | 0.0025 | 0.0023 | 0.0017 | 0.0018 | 0.0018 |  |
| Stom Lumen Mass (fbw) | SLc | 0.0098 | 0.0049 | 0.0024 | 0.0030 | 0.0030 |  |

**Table B.2 Human LADD Values Corresponding to Mouse POD Values**

|  |  |  | **BMDL/UFa*** | | | |
| --- | --- | --- | --- | --- | --- | --- |
|  |  |  | **Internal Dose (mg Cr(VI)/kg SI-day)** | | **External Dose (mg Cr(VI)/kg BW-day)** | |
| **Dose-Response Modeling (Software)** | **Response** | **BMR** | **Pyloric Flux** | **Total SI Flux** | **LADD Based on Pyloric Flux** | **LADD Based on Total SI Flux** |
| Benchmark Dose (BMDS) | Hyperplasia | 0.10 | 1.3 | 0.15 | 0.10 | 0.095 |
|  |  | 0.05 | 0.75 | 0.092 | 0.061 | 0.059 |
|  | Adenoma | 0.10 | 7.6 | 0.91 | 0.29 | 0.25 |
|  |  | 0.05 | 4.1 | 0.49 | 0.20 | 0.18 |
|  | Carcinoma | 0.10 | 20 | 2.4 | 0.52 | 0.43 |
|  |  | 0.05 | 15 | 1.8 | 0.44 | 0.37 |
| Constrained Nonlinear Regression (Graphpad Prism) | Hyperplasia | 0.10 | 0.87 | 0.11 | 0.07 | 0.069 |
|  |  | 0.05 | 0.49 | 0.061 | 0.041 | 0.040 |
|  | Adenoma | 0.10 | 6.7 | 0.80 | 0.27 | 0.24 |
|  |  | 0.05 | 3.8 | 0.46 | 0.19 | 0.17 |
|  | Carcinoma | 0.10 | 13 | 1.6 | 0.41 | 0.35 |
|  |  | 0.05 | 8.6 | 1.0 | 0.31 | 0.27 |

*UFa = 3

**Table B.3 Ex Vivo Data for the Reduction of Cr(VI) by Fasted Human Gastric Contents**

Fig B.1 Age differences in gastric pH behavior after a meal (A) and after PPI use in adults (B).

Fig. B.2. 24-Hour Time Profiles for GI Parameters and Small Intestines Flux Estimates: (A) Neonate; (B) Infant/Child; (C) Youth; (D) Adult; (E) Elderly; (F) Adult PPI User. Pyloric and SI flux estimates correspond to an oral dose of 0.0001 mg/kg-day spread over 4-6 exposure events (half during fed state, half during fasted state).

Fig. B.2 Cont’d. 24-Hour Time Profiles for GI Parameters and Small Intestines Flux Estimates: (A) Neonate; (B) Infant/Child; (C) Youth; (D) Adult; (E) Elderly; (F) Adult PPI User. Pyloric and SI flux estimates correspond to an oral dose of 0.0001 mg/kg-day spread over 4-6 exposure events (half during fed state, half during fasted state).

Fig. B.3. Age-Specific Contributions to Lifetime Average Daily Dose Values: Pyloric Flux (blue lines) and Small Intestines Flux (red lines). Example simulations for an oral dose of 0.0001 mg Cr(VI)/kg bw-day, with 50% of exposure occurring during fed state, and 50% of exposure occurring during fasted state (see lower panels of Figure B.2)

Figure B.4. Example Calculation for Human LADD for Mouse POD Value. The lowest data point for each curve reflects the time-weighted average depicted in Figure B.3. Simulations were repeated across a broad range of doses (up to 10 mg Cr(VI)/kg BW-day) to create these curves.

**B.5 Human Model Code Modified to Account for Diurnal Variation in Different Age Groups**

Program: Chromium Model

!PBPK model for chromium in humans

INITIAL

! Physiological parameters

! neonate, fed state = 6x2 hrs

TABLE PHSN, 1, 19 / 0.0, 0.5, 2.0, 4.0, 4.5, 6.0, 8.0, 8.5, 10., 12., 12.5, 14., 16., 16.5, 18., 20., 20.5, 22, 24, &

0., 1., 0., 0., 1., 0., 0., 1., 0., 0., 1.0, 0., 0., 1., 0., 0., 1., 0., 0. /

TABLE FEDN, 1, 24 / 0.0, 2.0, 2.01, 3.99, 4.0, 6.0, 6.01, 7.99, 8.0, 10.0, 10.01, 11.99, 12.0, 14., 14.01, 15.99, 16.0, 18.0, 18.01, 19.99, 20.0, 22.0, 22.01, 24., &

1., 1., 0., 0., 1., 1., 0., 0., 1., 1., 0., 0., 1., 1., 0., 0., 1., 1., 0., 0., 1., 1., 0., 0. /

! infant/child, fed state = 4x2 hrs

TABLE PHSC, 1, 14 / 0., 8., 8.5, 10., 11., 11.5, 13., 14., 14.5, 16., 18., 18.5, 20., 24., &

0., 0., 1., 0., 0., 1., 0., 0., 1., 0., 0., 1., 0., 0. /

TABLE FEDC, 1, 18 / 0., 7.99, 8., 10., 10.01, 10.99, 11., 13., 13.01, 13.99, 14., 16., 16.01, 17.99, 18., 20., 20.01, 24., &

0., 0., 1., 1., 0., 0., 1., 1., 0., 0., 1., 1., 0., 0., 1., 1., 0., 0. /

! youth & adult, fed state = 3x3 hrs

TABLE PHSA, 1, 23 / 0., 8., 8.5, 9., 9.5, 10., 10.5, 11., 12., 12.5, 13., 13.5, 14., 14.5, 15., 18., 18.5, 19., 19.5, 20., 20.5, 21., 24., &

0., 0., 1., 0.58, 0.33, 0.19, 0.11, 0., 0., 1., 0.58, 0.33, 0.19, 0.11, 0., 0., 1., 0.58, 0.33, 0.19, 0.11, 0., 0./

TABLE FEDA, 1, 14 / 0., 7.99, 8., 11., 11.01, 11.99, 12., 15., 15.01, 17.99, 18., 21., 21.01, 24., &

0., 0., 1., 1., 0., 0., 1., 1., 0., 0., 1., 1., 0., 0. /

! elderly, fed state = 3x4 hrs

TABLE PHSE, 1, 10 / 0., 8., 8.5, 12., 12.5, 16., 18., 18.5, 22., 24., &

0., 0., 1., 0., 1., 0., 0., 1., 0., 0. /

TABLE FEDE, 1, 10 / 0., 7.99, 8., 16., 16.01, 17.99, 18., 22., 22.01, 24., &

0., 0., 1., 1., 0., 0., 1., 1., 0., 0. /

! PPI adult, fed state = 3x3 hrs

TABLE PHSP, 1, 4 / 0., 6., 9., 24., &

0., 0., 1., 0./

TABLE FEDP, 1, 14 / 0., 7.99, 8., 11., 11.01, 11.99, 12., 15., 15.01, 17.99, 18., 21., 21.01, 24., &

0., 0., 1., 1., 0., 0., 1., 1., 0., 0., 1., 1., 0., 0. /

!Blood flows

CONSTANT QCC = 15.6 ! Cardiac output (L/h/kg)

CONSTANT QBC = 0.05 ! Fraction QCC, bone

CONSTANT QLC = 0.065 ! Fraction QCC, liver

CONSTANT QKC = 0.19 ! Fraction QCC, kidney

CONSTANT QPTC = 0.19 ! FRACTION QCC, PORTAL

CONSTANT QSC = 0.19 ! FRACTION QPTC, STOMACH

QSIC = 1-QSC ! FRACTION QPTC, SI

!Tissue volume

CONSTANT VBC = 0.14 ! Bone volume fraction in adult (L/kg)

CONSTANT VLC = 0.025 ! Liver volume fraction in adult (L/kg)

CONSTANT VKC = 0.0042 ! Kidney volume fraction in adult (L/kg)

CONSTANT VSC = 0.0018 ! Stomach Volume in adult (L/kg)

CONSTANT VSIC = 0.0078 ! SI VOLUME FRACTION

CONSTANT VBLC = 0.073 ! blood volume

CONSTANT HCT = 0.434 ! Hematocrit

CONSTANT FPT = 0.205 ! fraction blood volume in portal system

!LUMEN TO LUMEN TRANSIT (Cr & GI contents)

!CONSTANT KLSD = 1.9 ! LUMEN STOM TO LUMEN SI (/hr)

CONSTANT KLSDFED = 0.

CONSTANT KLSDFAST = 0.

CONSTANT KLSIL = 0.35 ! LUMEN SI TO LUMEN LI (/hr)

CONSTANT KFX = 0.034 ! L int to feces (/hr)

! lumen volumes (unitless fraction BW)

CONSTANT VSLC = 0.0034 ! stomach lumen (L/kg)

CONSTANT VSILC = 0.0039 ! lumen (L/kg)

!STOMACH UPTAKE

CONSTANT VMABS3S = 0. ! absorption in stom (mg/hr)

CONSTANT VMABS6S = 0. ! absorption in stom (mg/hr)

CONSTANT KMABS3S = 1000000. ! dose-dependent absorption of cr3, stomach (mg/L)

CONSTANT KMABS6S = 1000000. ! dose-dependent absorption of cr6, stomach (mg/L)

!SI UPTAKE

CONSTANT VMABS3 = 4.6 ! absorption in SI (mg/hr per cm)

CONSTANT KMABS3 = 1000000. ! dose-dependent absorption of cr6 (mg/L)

CONSTANT VMABS6 = 320. ! absorption in SI (mg/hr per cm)

CONSTANT KMABS6 = 1000000. ! dose-dependent absorption of cr6

CONSTANT KSLOUGH1 = 0. ! GI SLOUGHING (/hr); upper GI

CONSTANT KSLOUGH2 = 0.029 ! GI SLOUGHING (/hr); SI

CONSTANT LSI = 270 ! length SI (cm)

! CR6 REDUCTION

CONSTANT KRED = 71. ! All tissues/fluid except GI (/h)

CONSTANT KREDRC = 71. ! RBC (/h)

CONSTANT KREDBP = 0.66 ! Plasma (/h)

CONSTANT KMREDGIT = 0.000004 ! MM CONSTANT (MG)

CONSTANT VKREDGIT = 71 ! V/K RATIO FOR GIT REDUCTION (mg/hr)

VMREDGIT = VKREDGIT*KMREDGIT ! MAX RATE FOR GIT REDUCTION

CONSTANT KREDGIFC = 44.5 ! pH-scalable 2nd order rate constant based on modeling of ex vivo data (L/mg*hr)

KREDSIL = KREDGIFC*EXP(-PHSI) ! 2nd order rate constant for reduction in SI lumen conc (L/mg*hr)

!CONSTANT CRE0 = 20 ! Concentration of reducing equivalents in stomach contents based on modeling of ex vivo (mg/L gut contents)

CONSTANT CRE0FED = 0.

CONSTANT CRE0FAST = 0.

!CONSTANT PHS = 2.5 ! pH stomach lumen

CONSTANT PHPEAK = 5.

CONSTANT PHBASE = 1.7

CONSTANT PHSI = 6.5 ! pH SI lumen

!GI Fluid & Material Volume Rates

CONSTANT RFOODC = 0.0012 ! Rate of food consumption (L/hr*kg)

RFOOD = RFOODC*WBODY ! Rate of food consumption (L/hr)

CONSTANT RDRINKC = 0.00054 ! Rate of drinking water consumption in the adult rat (L/h*kg)

RDRINK = RDRINKC*WBODY ! Rate of drinking water consumption in the adult rat (L/h)

CONSTANT RGIFC = 0.0010 ! Rate of GI fluid production (L/hr*kg)

RGIF = RGIFC*WBODY ! Rate of GI fluid production (L/hr)

CONSTANT RSALC = 0.00063 ! Rate of Saliva production (L/hr*kg)

RSAL = RSALC*WBODY ! Rate of Saliva production (L/hr)

! TISSUE TRANSFER TO & FROM PLASMA; not scaled since multiplied by blood flows

CONSTANT KIN6 = 0.01 ! Cr6 from plasma to tissue (unitless)

CONSTANT KRBCIN6 = 0.15 ! Cr6 from plasma to tissue (L/hr)

CONSTANT KOUT6 = 0.0052 ! Cr6 from GI tissue to plasma (unitless)

CONSTANT KRBCIN3 = 0.0017 ! Cr3 from plasma to tissue (L/hr)

CONSTANT KRBCOUT3 = 0.0015 ! Cr3 from tissue to plasma (L/hr)

CONSTANT KINTCROTC = 0.0005 ! T-Cr from plasma to other tissue

CONSTANT KINTCRBC = 0.015 ! T-Cr from plasma to tissue

CONSTANT KINTCRLC = 0.0034 ! T-Cr from plasma to liver

CONSTANT KINTCRK = 0.005 ! T-Cr from plasma to kidney

CONSTANT KOUT3 = 0.0012 ! Cr3 from GI tissue to plasma

CONSTANT KOUTCCRGI = 0. ! C-Cr released from GI to portal blood

CONSTANT KOUTCCROTC = 0.0000083 ! C-Cr from tissue to plasma;

CONSTANT KOUTCCRLC = 0.000016 ! C-Cr from liver to plasma;

CONSTANT KOUTCCRBC = 0.00000024 ! C-Cr from bone to plasma;

CONSTANT KINCCR = 0.0015 ! C-Cr from plasma to kidney

CONSTANT SFIN = 0.15 ! Scaling factor (from mouse based on L:K ratios) for systemic tissue uptake Cr3

CONSTANT SFOUT = 40 ! Scaling factor (from mouse based on L:K ratios) for systemic tissue release Cr3

KINTCROT = KINTCROTC*SFIN

KINTCRB = KINTCRBC*SFIN

KINTCRL = KINTCRLC*SFIN

KOUTCCROT = KOUTCCROTC*SFOUT

KOUTCCRL = KOUTCCRLC*SFOUT

KOUTCCRB = KOUTCCRBC*SFOUT

! EXCRETION

CONSTANT KURCc = 0.03 ! Urinary excretion of CCR (/hr)

CONSTANT UrinCT = 24. ! Urine collection time (hrs), washout period for NTP studies

! EXPOSURE PARAMETERS

CONSTANT WATER3 = 0.0 ! Concentration of Cr3 in drinking water (mg/L)

CONSTANT WATER6 = 0.0 ! Concentration of Cr6 in drinking water (mg/L)

CONSTANT OD3 = 0.0 ! oral Cr3 dose (mg/kg)

CONSTANT OD6 = 0.0 ! oral Cr6 dose (mg/kg)

CONSTANT TINF = 0.05 ! time iv infusion (hr)

CONSTANT TEXPEND = 24 ! Time exposure ends

CONSTANT TSTOP = 48 ! Time simulation ends

CONSTANT DT1 = 0.

CONSTANT DT2 = 4.

CONSTANT DT3 = 8.

CONSTANT DT4 = 12.

CONSTANT DT5 = 16.

CONSTANT DT6 = 20.

CONSTANT DPCT1 = 1.0

CONSTANT DPCT2 = 0.

CONSTANT DPCT3 = 0.

CONSTANT DPCT4 = 0.

CONSTANT DPCT5 = 0.

CONSTANT DPCT6 = 0.

CONSTANT WADULT = 80 ! body weight

WBODY = WADULT

CONSTANT CRDOSE = 0. ! use for single dose studies

CONSTANT AGEGROUP = 3.

END !INITIAL

DYNAMIC

ALGORITHM IALG=2

CINTERVAL CINT=0.01

END !End dynamic

DERIVATIVE

IF(AGEGROUP.EQ.1) THEN !neonate

PHS = PHBASE + (PHPEAK-PHBASE)*PHSN(T)

KREDSL = KREDGIFC*EXP(-(PHBASE + (PHPEAK-PHBASE)*PHSN(T))) ! 2nd order rate constant for reduction in stomach lumen conc (L/mg*hr)

KLSD = KLSDFAST + (KLSDFED-KLSDFAST)*FEDN(T)

CRE0 = CRE0FAST + (CRE0FED-CRE0FAST)*FEDN(T)

ELSE

END IF

IF(AGEGROUP.EQ.2) THEN !infant & child

PHS = PHBASE + (PHPEAK-PHBASE)*PHSC(T)

KREDSL = KREDGIFC*EXP(-(PHBASE + (PHPEAK-PHBASE)*PHSC(T))) ! 2nd order rate constant for reduction in stomach lumen conc (L/mg*hr)

KLSD = KLSDFAST + (KLSDFED-KLSDFAST)*FEDC(T)

CRE0 = CRE0FAST + (CRE0FED-CRE0FAST)*FEDC(T)

END IF

IF(AGEGROUP.EQ.3) THEN !youth & adult

PHS = PHBASE + (PHPEAK-PHBASE)*PHSA(T)

KREDSL = KREDGIFC*EXP(-(PHBASE + (PHPEAK-PHBASE)*PHSA(T))) ! 2nd order rate constant for reduction in stomach lumen conc (L/mg*hr)

KLSD = KLSDFAST + (KLSDFED-KLSDFAST)*FEDA(T)

CRE0 = CRE0FAST + (CRE0FED-CRE0FAST)*FEDA(T)

END IF

IF(AGEGROUP.EQ.4) THEN !elderly

PHS = PHBASE + (PHPEAK-PHBASE)*PHSE(T)

KREDSL = KREDGIFC*EXP(-(PHBASE + (PHPEAK-PHBASE)*PHSE(T))) ! 2nd order rate constant for reduction in stomach lumen conc (L/mg*hr)

KLSD = KLSDFAST + (KLSDFED-KLSDFAST)*FEDE(T)

CRE0 = CRE0FAST + (CRE0FED-CRE0FAST)*FEDE(T)

END IF

IF(AGEGROUP.EQ.5) THEN !PPI user

PHS = PHBASE + (PHPEAK-PHBASE)*PHSP(T)

KREDSL = KREDGIFC*EXP(-(PHBASE + (PHPEAK-PHBASE)*PHSP(T))) ! 2nd order rate constant for reduction in stomach lumen conc (L/mg*hr)

KLSD = KLSDFAST + (KLSDFED-KLSDFAST)*FEDP(T)

CRE0 = CRE0FAST + (CRE0FED-CRE0FAST)*FEDP(T)

END IF

Day = t/24

!SCALED FLOW RATES

QC = QCC*WADULT**0.74 ! Cardiac output in the adult (L/hr)

QCG = (QC*((WBODY/WADULT)**0.67))*(1-HCT) ! Cardiac plasma output as a function of age-specific bw (L/h)

QL = QLC*QC ! plasma flow to liver (L/h)

QK = QKC*QCG ! plasma flow to kidney (L/h)

QB = QBC*QCG ! plasma flow to bone (L/h)

QRBC = (QC*((WBODY/WADULT)**0.67))*(HCT) ! RBC flow in systemic blood

QPT = QPTC*QCG ! Fraction QCC, PORTAL

QS = QSC*QPT ! plasma flow to STOM (L/h)

QSI = QSIC*QPT ! plasma flow to DUOD (L/h)

QPTRBC = QPT*HCT/(1-HCT) ! rbc flow in portal system

QOT = QCG-(QL+QK+QB+QPT) ! plasma flow to other tissues

! TISSUE VOLUMES (L)

VL = VLC*WBODY ! liver volume (L)

VK = VKC*WBODY ! kidney volume (L)

VS = VSC*WBODY ! stomach volume (L)

VSI = VSIC*WBODY ! SI volume (L)

VB = VBC*WBODY ! Bone volume (L)

VBL = VBLC*WBODY ! Blood volume (L)

VRBC = VBL*HCT ! Red blood cell volume (L)

VRBCSYS = VRBC*(1-FPT) ! volume rbc in systemic blood (L)

VRBCPT = VRBC*FPT ! volume roc in portal blood (L)

VBP = VBL*(1-HCT) ! Blood plasma volume (L)

VBPSYS = VBP*(1-FPT) ! volume plasma in systemic blood (L)

VBPPT = VBP*FPT ! volume plasma in portal blood (L)

VOT = WBODY-VB-VL-VK-VS-VSI-VBL ! Volume of other tissue (L)

! Lumen Volumes (L)

VSL = VSLC*WBODY

VSIL = VSILC*WBODY

! ===================================================================================

KURC = KURCc*WBODY**0.75 ! Urinary Excretion Cr3, storage/excretion pool

!DOSING RATES

INGST3 = WATER3*RDRINK ! Rate of ingestion of Cr3 in the adult rat (mg/h)

INGST6 = WATER6*RDRINK ! Rate of ingestion of Cr6 in the adult rat (mg/h)

! On/off switch for Cr(III & VI) exposure (mg/h)

IF (t.LE.Texpend) THEN

DW3 = INGST3

DW6 = INGST6

OAMT31 = OD3*WBODY*PULSE(DT1,24,TINF)*DPCT1

OAMT32 = OD3*WBODY*PULSE(DT2,24,TINF)*DPCT2

OAMT33 = OD3*WBODY*PULSE(DT3,24,TINF)*DPCT3

OAMT34 = OD3*WBODY*PULSE(DT4,24,TINF)*DPCT4

OAMT35 = OD3*WBODY*PULSE(DT5,24,TINF)*DPCT5

OAMT36 = OD3*WBODY*PULSE(DT6,24,TINF)*DPCT6

OAMT61 = OD6*WBODY*PULSE(DT1,24,TINF)*DPCT1

OAMT62 = OD6*WBODY*PULSE(DT2,24,TINF)*DPCT2

OAMT63 = OD6*WBODY*PULSE(DT3,24,TINF)*DPCT3

OAMT64 = OD6*WBODY*PULSE(DT4,24,TINF)*DPCT4

OAMT65 = OD6*WBODY*PULSE(DT5,24,TINF)*DPCT5

OAMT66 = OD6*WBODY*PULSE(DT6,24,TINF)*DPCT6

ORATE6 = (OAMT61+OAMT62+OAMT63+OAMT64+OAMT65+OAMT66)/TINF !oral amount (mg)

ORATE3 = (OAMT31+OAMT32+OAMT33+OAMT34+OAMT35+OAMT36)/TINF !oral amount (mg)

ELSE

DW3=0.0

DW6=0.0

ORATE6 = 0.

ORATE3 = 0.

ENDIF

RING6 = DW6+ORATE6 ! Rate Cr6 ingested (mg/hr)

RING3 = DW3+ORATE3 ! Rate Cr3 ingested (mg/hr)

AING6 = INTEG(RING6,0.) ! Amount Cr6 ingested (mg)

AING3 = INTEG(RING3,0.) ! Amount Cr3 ingested (mg)

AING = AING6 + AING3 ! Total amount ingested (mg)

! ********************** Stomach **********************

! Stomach Lumen

RRESL = RREPROD - RREDSL - RTRANSS ! Rate of change in stomach reducing equivalents (mg/hr)

ARESLA = INTEG(RRESL, 0.) ! Amount of reducing equivalents (mg)

ARESL = MAX(ARESLA,0.)

RREPROD = CRE0*(RGIF+RDRINK+RFOOD+RSAL) ! rate reducing equivalents produced (mg/hr)

RTRANSS = KLSD*ARESL ! rate reducing equivalents transit to SI (mg/hr)

CRESL = ARESL/VSL ! Concentration of reducing equivalents in stomach lumen (mg/L)

RSL6 = RING6-RREDSL-RABSS6-R2D6+RSLFS6 ! Rate change in stomach lumen Cr6 (mg/hr)

ASL6A = INTEG(RSL6,0.0) ! amount in stomach lumen (mg)

ASL6 = MAX(ASL6A,0.)

RREDSL = CSL6*KREDSL*CRESL*VSL ! reduction rate (mg/hr)

RABSS6 = VMABS6S*CSL6/(KMABS6S+CSL6) ! absorption rate Cr6 (mg/hr)

ABSS6 = INTEG(RABSS6, 0.) ! amount Cr6 absorbed (mg)

R2D6 = KLSD*ASL6 ! rate transfer to SI (mg/hr)

A2D6 = INTEG(R2D6, 0.) ! amt cr6 transferred to SI per L tissue (mg/L)

AMT2D6 = A2D6/(VSI) ! amt cr6 transferred to SI per L tissue (mg/L)

CSL6 = ASL6/VSL ! concentration Cr6 in stomach lumen (mg/L)

RSL3 = RING3+RREDSL-RABSS3-R2D3+RSLFS3 ! Rate change in stomach lumen Cr3 (mg/hr)

ASL3A = INTEG(RSL3,0.) ! amount in stomach lumen (mg)

ASL3 = MAX(ASL3A,0.)

RABSS3 = VMABS3S*CSL3/(KMABS3S+CSL3) ! absorption rate (mg/hr)

ABSS3 = INTEG(RABSS3, 0.) ! amount Cr3 absorbed (mg)

R2D3 = KLSD*ASL3 ! rate transit to SI (mg/hr)

A2D3 = INTEG(R2D3, 0.) ! amt cr3 transferred to SI (mg)

AMT2D3 = A2D3/(VSI) ! amt cr3 transferred to SI per L tissue (mg/L)

CSL3 = ASL3/VSL ! concentration of Cr3 in stomach lumen (mg/L)

ASL = ASL3 + ASL6 ! total amount Cr in stom lumen (mg)

CSL = ASL/VSL ! concentration Cr in stom lumen (mg/L)

! Stomach Tissue

RS6 = RABSS6 - RREDS - RS6OUT - RSLFS6 ! rate change in stomach epith (mg/hr)

AS6A = INTEG(RS6,0.) ! amount in stomach epith (mg)

AS6 = MAX(AS6A,0.)

CS6 = AS6/VS ! concentration in stomach epith (mg/L)

RS6OUT = KOUT6*CS6*QS ! rate Cr6 to plasma (mg/hr)

RREDS = CS6*VMREDGIT/(KMREDGIT+CS6)

RSLFS6 = AS6*KSLOUGH1 ! cell sloughing (mg/hr)

RS3 = RABSS3 + RREDS - RS3OUT - RSOUTTCR - RSLFS3 ! rate change in stomach epith (mg/hr)

AS3A = INTEG(RS3,0.) ! amount in stomach epith (mg)

AS3 = MAX(AS3A,0.)

CS3 = AS3/VS ! concentration in stomach epith (mg/L)

RSOUTTCR = KOUT3*CS3*QS ! rate Cr3 to plasma as T-Cr (mg/hr)

RS3OUT = KOUTCCRGI*CS3*QS ! rate C-Cr to plasma (mg/hr)

RSLFS3 = AS3*KSLOUGH1 ! cell sloughing

AS = AS3 + AS6 ! total amount (mg)

CS = AS/VS ! total concentration (mg)

!***************** S. INTESTINE ********************

! SI Lumen

RRESIL = RTRANSS - RREDSIL - RTRANSSI ! Rate of change in SI reducing equivalents (mg/hr)

ARESILA = INTEG(RRESIL, 0.) ! Amount of reducing equivalents (mg)

ARESIL = MAX(ARESILA,0.)

RTRANSSI = KLSIL*ARESIL ! rate reducing equivalents transit to LI (mg/hr)

CRESIL = ARESIL/VSIL ! Concentration of reducing equivalents in SI lumen (mg/L)

RSIL6 = R2D6 - RREDSIL - RABSSI6 - R2LI6 ! rate change in Cr6 SI lumen (mg/hr)

ASIL6a = INTEG(RSIL6,0.) ! amount Cr6 (mg)

ASIL6 = MAX(ASIL6A,0.)

RREDSIL = CSIL6*KREDSIL*CRESIL*VSIL ! rate reduction (mg/hr)

RABSSI6 = LSI*(VMABS6*CSIL6/(KMABS6+CSIL6)) ! rate Cr6 absorption (mg/hr)

ABSSI6 = INTEG(RABSSI6, 0.) ! amount Cr6 absorbed (mg)

AMTABSSI6 = ABSSI6/VSI ! amount Cr6 absorbed per L tissue (mg/L)

R2LI6 = KLSIL*ASIL6 ! rate transit to LI (mg/hr)

CSIL6 = ASIL6/VSIL ! concentration Cr6 in SI lumen (mg/L)

RSIL3 = R2D3 + RREDSIL - RABSSI3 - R2LI3 ! rate change in Cr3 SI lumen (mg/hr)

ASIL3A = INTEG(RSIL3,0.) ! amount Cr3 (mg)

ASIL3 = MAX(ASIL3A,0.)

RABSSI3 = LSI*(VMABS3*CSIL3/(KMABS3+CSIL3)) ! rate absorption (mg/hr);

ABSSI3 = INTEG(RABSSI3, 0.) ! amount Cr3 absorbed (mg)

AMTABSSI3 = ABSSI3/VSI ! amount Cr6 absorbed per L tissue (mg/L)

R2LI3 = KLSIL*ASIL3 ! rate transit to LI (mg/hr)

CSIL3 = ASIL3/VSIL ! concentration Cr3 in SI lumen (mg/L)

ASIL = ASIL3 + ASIL6 ! total Cr amount (mg)

CSIL = ASIL/VSIL ! total Cr conc (mg/L)

! SI Tissue

RSI6 = RABSSI6 - RREDSI - RSI6OUT - RSLFSI6 ! rate change in Cr6 SI epith (mg/hr)

ASI6A = INTEG(RSI6,0.) ! amount Cr6 (mg)

ASI6 = MAX(ASI6A,0.)

CSI6 = ASI6/VSI ! concentration Cr6 (mg/L)

RREDSI = CSI6*VMREDGIT/(KMREDGIT+CSI6) ! rate reduction (mg/hr)

AREDSI = INTEG(RREDSI, 0.) ! amount reduced (mg)

AMTREDSI = AREDSI/VSI ! amount reduced per L tissue (mg/L)

RSI6OUT = KOUT6*CSI6*QSI ! rate out to plasma (mg/hr)

RSLFSI6 = KSLOUGH2*ASI6 ! rate SI slough to lumen (mg/hr)

RSI3 = RABSSI3 + RREDSI - RSI3OUT - RSIOUTTCR - RSLFSI3 ! rate change in Cr3 SI epith (mg/hr)

ASI3A = INTEG(RSI3,0.) ! amount Cr3 (mg)

ASI3 = MAX(ASI3A,0.)

CSI3 = ASI3/VSI ! concentration Cr3 (mg/L)

RSLFSI3 = KSLOUGH2*ASI3 ! rate sloughed (mg/hr)

RSIOUTTCR = KOUT3*CSI3*QSI ! rate Cr3 out to plasma as T-Cr (mg/hr)

RSI3OUT = KOUTCCRGI*CSI3*QSI ! rate C-Cr out to plasma (mg/hr)

ASI = ASI3 + ASI6 ! total Cr amount (mg)

CSI = ASI/VSI ! total Cr concentration (mg/L)

AMTABS6 = ABSSI6/VSI ! amount Cr6 absorbed in SI per L SI (mg/L)

AMTABS3 = ABSSI3/VSI ! amount Cr3 absorbed in SI per L SI (mg/L)

! ABSORPTION IN LG INTESTINE ASSUMED TO BE NEGLIGIBLE; COMPARTMENT ADDED FOR TRANSIT TO MATCH FECAL EXCRETION DATA

! LG Intestine lumen, nonphysiologic

RLIL6 = R2LI6-RFX6 ! rate of change Cr6 in LI lumen (mg/hr)

ALIL6 = INTEG(RLIL6, 0.) ! amount Cr6 LI lumen (mg)

RFX6 = KFX*ALIL6 ! rate Cr6 fecal excretion (mg/hr)

AFX6 = INTEG(RFX6, 0.) ! amount Cr6 excreted in feces (mg)

RLIL3 = R2LI3-RFX3 ! rate of change Cr3 in LI lumen (mg/hr)

ALIL3 = INTEG(RLIL3, 0.) ! amount Cr3 LI lumen (mg)

RFX3 = KFX*ALIL3 ! rate Cr3 fecal excretion (mg/hr)

AFX3 = INTEG(RFX3, 0.) ! amount Cr3 excreted in feces (mg)

ALIL = ALIL6+ALIL3

!---------------------------------------------

!***************** PORTAL BLOOD ********************

! Portal Plasma

RPT6IN = KRBCIN6*CPT6 ! rate Cr6 from plasma to RBC (mg/hr)

RREDPTP = KREDBP*APT6 ! rate reduced (mg/hr)

RSYS2PT6 = CBP6*QPT ! rate Cr6 in from systemic plasma (mg/hr)

RPT2SYS6 = CPT6*QPT! ! rate Cr6 out to systemic plasma (mg/hr)

CPT6=((RS6OUT+RSI6OUT)+RSYS2PT6)/(QPT+KRBCIN6+KIN6*QPT+KREDBP*VBPPT)

APT6=CPT6*VBPPT

RPT3IN = KRBCIN3*CPT3 ! rate to RBC (mg/hr)

RPT3OUT = KRBCOUT3*CPTRBC3 ! rate from RBC (mg/hr)

RSYS2PT3 = CTCRP*QPT ! rate Cr3 in from systemic plasma (mg/hr)

RPT2SYS3 = CPT3*QPT ! rate Cr3 out to systemic plasma (mg/hr)

CPT3=((RSOUTTCR+RSIOUTTCR)+RSYS2PT3+RREDPTP)/(QPT+KRBCIN3+KINTCRL*QPT)

APT3=CPT3*VBPPT

CPTCCR=((RS3OUT+RSI3OUT+RPT3OUT)+QPT*CCCRP)/QPT

APTCCR=CPTCCR*VBPPT

! Portal RBC

RREDPTC = KREDRC*APTRBC6 ! rate reduced (mg/hr)

CPTRBC6=(QPTRBC*CRBC6+RPT6IN)/(QPTRBC+KREDRC*VRBCPT)

APTRBC6=CPTRBC6*VRBCPT

CPTRBC3=(QPTRBC*CRBC3+RPT3IN+RREDPTC)/(QPTRBC+KRBCOUT3)

APTRBC3=VRBCPT*CPTRBC3

!********************* LIVER ***********************

RL6 = RL6IN+RL6INPT-RREDLIV ! Rate of change of Cr6 liver (mg/hr)

AL6A = INTEG(RL6,0.) ! Amount Cr6 (mg)

AL6 = MAX(AL6A,0.)

CL6 = AL6/VL ! Concentration Cr6 (mg/L)

RL6IN = KIN6*CBP6*QL ! Rate Cr6 from systemic plasma (mg/hr)

RL6INPT = KIN6*CPT6*QPT ! rate T-Cr in from portal plasma (mg Cr/hr)

RREDLIV = KRED*AL6 ! Rate of reduction of Cr6 to Cr3 (mg/h)

RL3 = RL3IN+RL3INPT+RREDLIV-RL3OUT ! rate of T-Cr in liver (mg Cr/hr)

AL3A = INTEG(RL3, 0.) ! mass of T-Cr in liver (mg Cr/L)

AL3 = MAX(AL3A,0.)

CL3 = AL3/VL ! conc of T-Cr in liver (mg Cr/L)

RL3IN = CTCRP*KINTCRL*QL ! rate T-Cr in from systemic plasma (mg Cr/hr)

RL3INPT = CPT3*KINTCRL*QPT ! rate T-Cr in from portal plasma (mg Cr/hr)

RL3OUT = KOUTCCRL*CL3*(QL+QPT) ! rate CCr released to plasma (mg Cr/hr)

AL = AL6 + AL3 ! Amount of chromium in liver (mg)

CL = AL/VL ! Total concentration in liver (mg/L)

!*************** SYSTEMIC PLASMA ***************************

!Three forms of Cr in plasma: Cr6, T-Cr=distribution pool Cr3, C-Cr=storage/excretion pool Cr3

RBP6 = (RPT2SYS6-RSYS2PT6)-RL6IN-RK6IN-RB6IN-ROT6IN-RIN6-RREDP ! rate change Cr6 plasma (mg/hr)

ABP6A = INTEG(RBP6, 0.) ! amount Cr6 (mg)

ABP6 = MAX(ABP6A,0.)

CBP6 = ABP6/VBPSYS ! concentration Cr6 (mg/L)

RREDP = KREDBP*ABP6 ! rate reduced (mg/hr)

RTCRP = (RPT2SYS3-RSYS2PT3)-RL3IN-RK3IN-RIN3-ROT3IN-RB3IN+RREDP ! rate change in plasma T-Cr (mg/hr)

ATCRPA = INTEG(RTCRP, 0.) ! amount T-Cr (mg Cr)

ATCRP = MAX(ATCRPA,0.)

CTCRP = ATCRP/VBP ! concentration T-Cr (mg Cr/L)

RCCRP = (RL3OUT+ROUT3+ROT3OUT+RB3OUT)-RKCCRIN+QPT*(CPTCCR-CCCRP) ! Rate change in plasma C-Cr (mg/hr)

ACCRPA = INTEG(RCCRP, 0.) ! amount C-Cr (mg Cr)

ACCRP = MAX(ACCRPA,0.)

CCCRP = ACCRP/VBPSYS ! concentration C-Cr (mg Cr)

ABP = ABP6 + ATCRP + ACCRP ! Concentration of chromium in plasma (mg/L)

CBP = ABP/VBPSYS ! Total amount of chromium in plasma (mg)

ABL = ABP+ARBC ! Total amount of chromium in blood (mg)

CBL = ABL/VBL ! Concentration of chromium in blood (mg/L)

!******************* Systemic RBC ***********************

RRBC6 = QPTRBC*(CPTRBC6-CRBC6)+RIN6-RREDRC ! rate of change Cr6 in RBC (mg/hr)

ARBC6A = INTEG(RRBC6,0.) ! Amount Cr6 (mg)

ARBC6 = MAX(ARBC6A,0.)

CRBC6 = ARBC6/VRBCSYS ! Concentration Cr6 (mg/L)

RIN6 = KRBCIN6*CBP6 ! Rate Cr6 in from plasma (mg/hr)

RREDRC = KREDRC*ARBC6 ! Rate reduced (mg/hr)

RRBC3 = QPTRBC*(CPTRBC3-CRBC3)+(RIN3-ROUT3)+RREDRC ! rate of change Cr3 in RBC (mg/hr)

ARBC3A = INTEG(RRBC3,0.) ! Amount of Cr3 (mg)

ARBC3 = MAX(ARBC3A,0.)

CRBC3 = ARBC3/VRBCSYS ! Concentration Cr3 (mg/L)

RIN3 = KRBCIN3*CTCRP ! Rate in from plasma (mg/hr)

ROUT3 = KRBCOUT3*CRBC3 ! Rate out to plasma (mg/hr)

ARBC = (ARBC3+ARBC6)+(APTRBC3+APTRBC6) ! Total amount of Cr in systemic & portal RBC (mg)

CRBC = ARBC/VRBC ! Total concentration of chromium in RBC (mg/L)

RBC2P = (CRBC3+CRBC6)/(CBP+0.000000001)

!**************************** KIDNEY *********************************

RK6 = RK6IN-RREDK ! Rate of change in Cr6 kidney (mg/hr)

AK6A = INTEG(RK6,0.) ! Amount of Cr6 (mg)

AK6 = MAX(AK6A,0.)

CK6 = AK6/VK ! Concentration Cr6 (mg/L)

RK6IN = KIN6*CBP6*QK ! rate in from plasma (mg/hr)

RREDK = KRED*AK6 ! Rate of reduction (mg/hr)

RK3 = RK3IN+RREDK+RKCCRIN-RURC ! Rate of change in Cr3 kidney (mg/hr)

AK3A = INTEG(RK3, 0.) ! Amount Cr (mg)

AK3 = MAX(AK3A,0.)

CK3 = AK3/VK ! Concentration Cr (mg Cr/L)

RK3IN = CTCRP*KINTCRK*QK ! Rate T-CR from plasma (mg/hr), assumed converted to C-CR for excretion

RKCCRIN = CCCRP*KINCCR*QK ! Rate C-CR from plasma (mg/hr)

RURC = KURC*CK3 ! Urinary Excretion C-Cr

AK = AK3+AK6 ! Amount of Cr in kidney (mg)

CK = AK/VK ! Concentration Cr in kidney (mg/L)

IF(CL*CK.GT.0) LKRatio = CL/CK ! ratio of liver:kidney concentration

!*********************** OTHER TISSUES ******************************

ROT6 = ROT6IN-RREDOT ! Rate of change in Cr6 other tissues (mg/h)

AOT6A = INTEG(ROT6,0.) ! Amount of Cr6 (mg)

AOT6 = MAX(AOT6A,0.)

COT6 = AOT6/VOT ! Concentration Cr6 (mg/L)

ROT6IN = KIN6*CBP6*QOT ! Rate Cr6 in from plasma (mg/hr)

RREDOT = KRED*AOT6 ! Rate of reduction (mg/hr)

ROT3 = ROT3IN - ROT3OUT + RREDOT ! Rate of change in Cr3 (mg/hr)

AOT3A = INTEG(ROT3,0.) ! Amount of Cr3 (mg)

AOT3 = MAX(AOT3A,0.)

COT3 = AOT3/VOT ! Concentration of Cr3 (mg/L)

ROT3IN = CTCRP*KINTCROT*QOT ! rate T-Cr in from plasma (mg/hr)

ROT3OUT = COT3*KOUTCCROT*QOT ! rate C-Cr released to plasma (mg/hr)

AOT = AOT6+AOT3 ! Total amount of chromium in other tissues (mg)

COT = AOT/VOT ! Total concentration in other tissues (mg/L)

!*************************** BONE *********************************

RB6 = RB6IN-RREDB ! Rate of change in Cr6 bone (mg/hr)

AB6A = INTEG(RB6,0.) ! Amount of Cr6 (mg)

AB6 = MAX(AB6A,0.)

CB6 = AB6/VB ! Concentration Cr6 (mg/L)

RB6IN = KIN6*CBP6*QB ! rate in from plasma (mg/hr)

RREDB = KRED*AB6 ! Rate of reduction (mg/hr)

RB3 = RB3IN+RREDB-RB3OUT ! Rate of change in Cr3 bone (mg/hr)

AB3A = INTEG(RB3,0.) ! Amount Cr3 (mg)

AB3 = MAX(AB3A,0.)

CB3 = AB3/VB ! Concentration Cr3 (mg/L)

RB3IN = CTCRP*KINTCRB*QB ! rate T-Cr in from plasma (mg/hr)

RB3OUT = CB3*KOUTCCRB*QB ! rate C-Cr released to plasma (mg/hr)

AB = AB3+AB6 ! Total Cr in bone (mg)

CB = AB/VB ! Total Cr concentration in bone (mg/L)

!Urinary Excretion

AURX = INTEG(RURC,0.) ! Total amount of chromium in urine (mg)

AURXDELAY = DELAY(AURX, 0, UrinCT, 5000, 1) ! Delay for calculating excretion over collection time

AURXPERDAY = AURX-AURXDELAY ! Cr excreted in urine over collection time (mg)

IF(AING.GT.0) FracABS = (BB+AURX)/AING ! Fraction Cr absorbed

!Fecal Excretion

RFX = RFX3+RFX6+RSLFSI3+RSLFSI6 ! Total rate Cr excreted in feces (mg/hr)

AFX = INTEG(RFX, 0.) ! total amount Cr excreted in feces (mg)

! Total excretion

RX = RURC + RFX ! Total rate of excretion of Cr (mg/day)

AX = AURX + AFX ! Total amount of Cr excreted (mg)

! Mass balance

FLOWCHECK = QCG-(QL+QK+QOT+QPT+QB) ! plasma flow (L/hr)

VOLUMECHECK = WBODY-(VL+VK+VS+VSI+VB+VOT+VBL) ! tissue volume (L)

BB6 = AL6+AK6+AS6+ASI6+AOT6+AB6+ARBC6+ABP6+APT6+APTRBC6 ! amount (mg)

BB3 = AL3+AK3+AS3+ASI3+AOT3+AB3+ARBC3+APTRBC3+ATCRP+APT3+ACCRP+APTCCR ! amount (mg)

Lumen = ASL3+ASL6+ASIL3+ASIL6+ALIL3+ALIL6 ! amount (mg)

BB = BB3+BB6 ! amount (mg)

BBLumen = BB+Lumen ! amount (mg)

MASSCHECK = AING-BB-AX-Lumen ! amount (mg)

TERMT(T.GT.Tstop)

END !DERIVATIVE

END !PROGRAM
